# Supplementary figures and images for: New insights into the role of microheterogeneity of ZP3 during structural maturation of the avian equivalent of mammalian zona pellucida
Source: PLoS One. 2023 Mar 21;18(3):e0283087. doi: 10.1371/journal.pone.0283087 (PMC10030024; doi:10.1371/journal.pone.0283087)

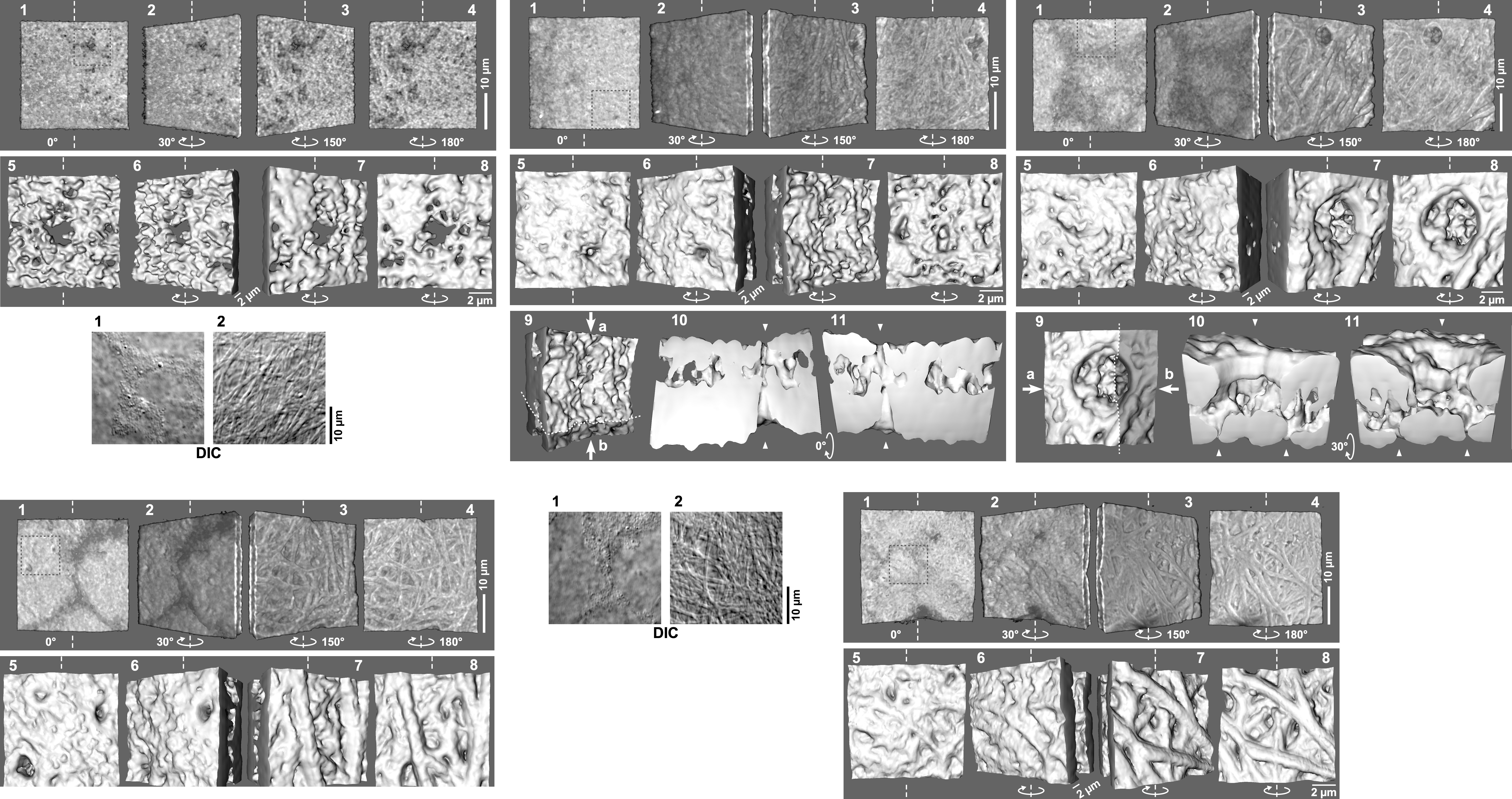

Supplement: S1 Fig — Other images for 3D-surface morphologies of egg-coat matrices in the F3 (A), F2 (B, C and E), and F1 (G) IPVLs are shown in the same format as Fig 1, except that the look-down angles of panels 10 and 11 in both B and C are 0° and 30° (indicated by white elliptic arrows), respectively, and that panels 9–11 that were shown in Fig 1B are not contained in E. In addition, Differential-interference-contrast (DIC) microscopy images of the 30-μm square areas for the F2 IPVLs (D and F) were obtained as the controls in focus on near the smooth surfaces (panels 1) and on the fibrous surfaces (panels 2), respectively. Bar: 10 μm. (TIF) [file pone.0283087.s001.tif]

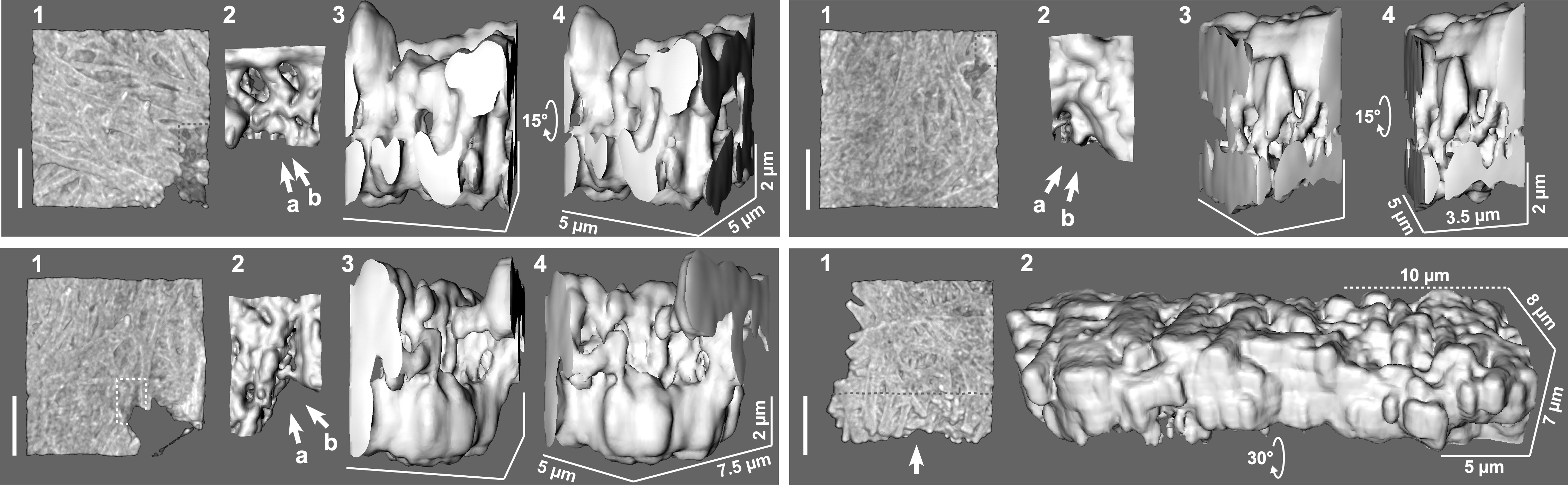

Supplement: S2 Fig — Other images for inner structures of egg-coat matrices in the F2 (A–C) and F1 (D) IPVLs are shown in the similar format as Fig 2, except that the areas shown by gray dotted lines inside the 30-μm square images for egg-coat matrices in the F2 IPVLs (panels 1 in A–C) were rendered as the magnified 3D-surface images from the viewpoints shown by white arrows a and b in panels 2 with look-down angles of 15° indicated by white elliptic arrows (panels 3 and 4 in both A and B) and 0° (panels 3 and 4 in C). (TIF) [file pone.0283087.s002.tif]

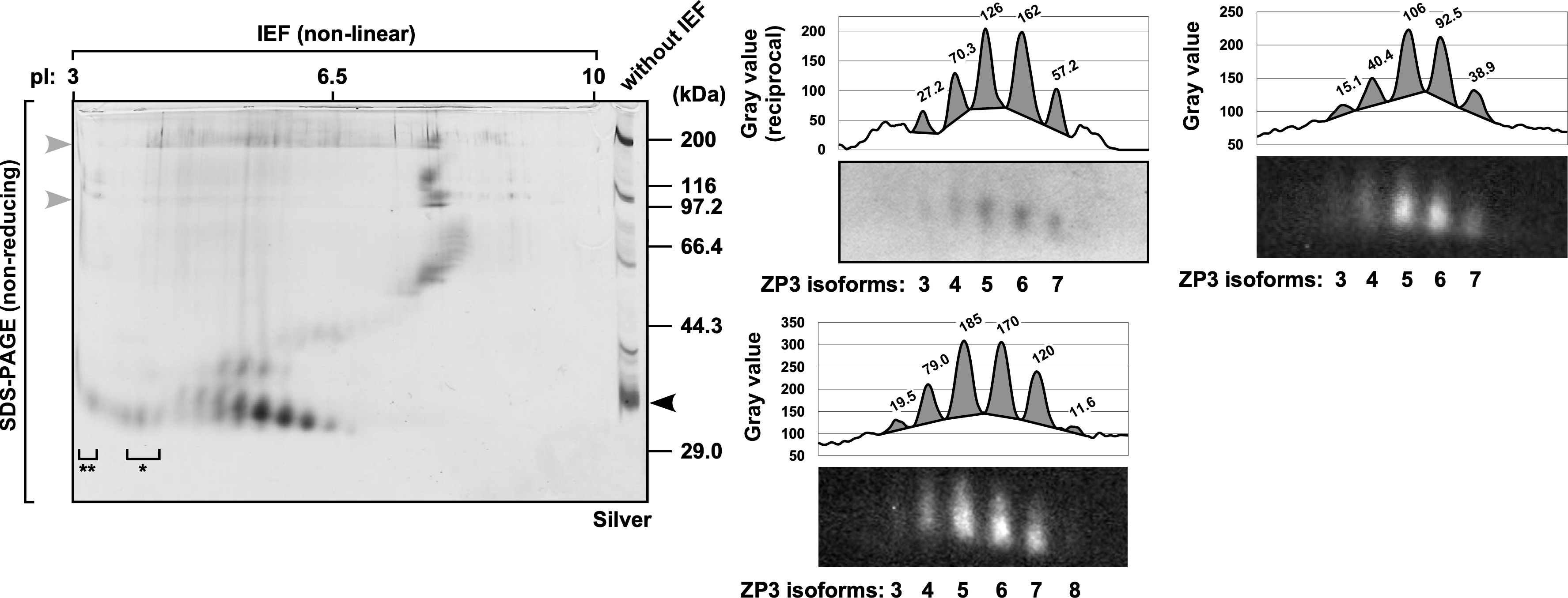

Supplement: S3 Fig — (A) The original gel image of the panel 1 in Fig 3A. For comparison, proteins in the F1 IPVL of the commercial White-Leghorn hen were separated by non-reducing SDS-PAGE without IEF (right side lane). Migration positions of the disulfide-linked dimer and the monomer of ZP1 (upper and lower gray arrowheads, respectively) are shown on the left side of the gel image, and that of ZP3 (black arrowhead) and the MW markers are shown on the right side of it. Single and double asterisks are unidentified proteins also detected in our previous study [25]. (B–E) Results of densitometric analyses (upper panels) against ZP3 spot images (lower panels being identical to panels 2–5 in Fig 3A, respectively, although all are ranging from pI ~4–7) are shown. The vertically averaged pixel intensities (the gray values) were plotted against the horizontal distances, and the peak areas were measured using the tangent-skim method, although the values are reciprocal only in B. The measured peak areas are shown in the plots, and the positions of the corresponding ZP3 isoforms are shown on the bottom. (TIF) [file pone.0283087.s003.tif]

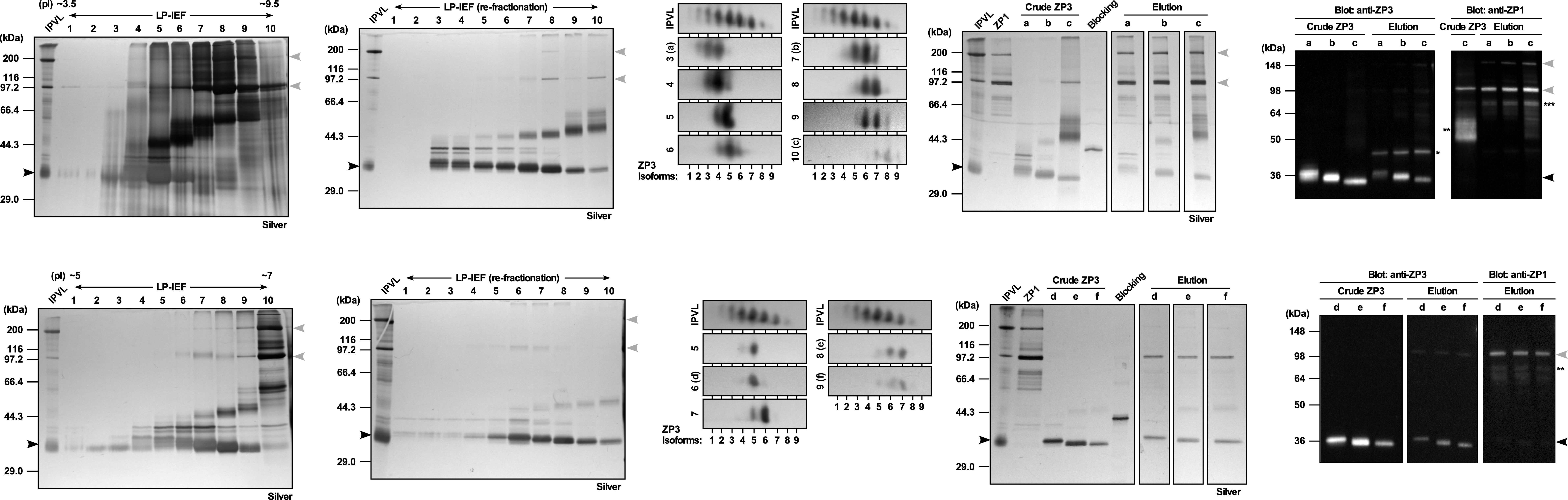

Supplement: S5 Fig — (A and F) Non-reducing SDS-PAGE gel images of each 10 fractions collected from the first step of the LP-IEFs before re-fractionation procedures using carrier ampholytes for pH ranges of ~3.5–9.5 and ~5–7, respectively, followed by silver staining. The pH ranges and fraction numbers are shown on the top of the images, and IPVLs were subjected to the left-side lanes of fractions 1 as controls. Migration positions of the disulfide-linked dimer and the monomer of ZP1 (upper and lower gray arrowheads, respectively) are shown on the right side, and that of ZP3 (black arrowhead) and the MW markers are shown on the left side of the gel images. The fractions 5 and 6 seen in A and the fractions 7 to 9 that are seen in F were pooled to be subjected to the subsequent re-fractionation procedure, respectively. (B and G) The original gel images of Fig 6A and 6E. Migration positions of ZP1 dimer and monomer, ZP3, and the MW markers are shown similarly to A. (C and H) The silver-stained 2-D gel images of all the ZP3 isoform-containing fractions 3–10 and 5–9 that are seen in Fig 6A and 6E, respectively, are extracted similarly to the Fig 6B and 6F. The extracted 2-D gel images of IPVLs are shown on the top as controls, and the horizontal positions of the spots of ZP3 isoforms are indicated on the bottom with vertical lines. (D and I) Silver-stained non-reducing SDS-PAGE gel images of both the crude ZP3-isoform fractions (labeled as "Crude ZP3") and the elution fractions (labeled as "Elution") for the fractions a–c and d–f in Fig 6B and 6F, respectively. IPVLs, the ZP1 solution and the His6-tagged thioredoxin (for blocking agent) that were used to prepare the ZP1-immobilized beads were also subjected. Migration positions of ZP1 dimer and monomer, ZP3, and the MW markers are shown similarly to A. (E and J) The original blot images of Fig 6C and 6G, although including the result of immunoblotting for the crude ZP3-isoform fraction c using anti-ZP1 antiserum. Migration positions of ZP1 d [file pone.0283087.s005.tif]
