# Supplementary material for: New insights into the role of microheterogeneity of ZP3 during structural maturation of the avian equivalent of mammalian zona pellucida
Source: PLoS One. 2023 Mar 21;18(3):e0283087. doi: 10.1371/journal.pone.0283087 (PMC10030024; doi:10.1371/journal.pone.0283087)
Supplement: S1 Raw images — (PDF) [file pone.0283087.s007.pdf]

Original gel image for panel 1 of Fig3A and S3A Fig

• The MW labeling depended on the comparison of these lanes.

• The pI labeling depended on pH range of the used gel strip.

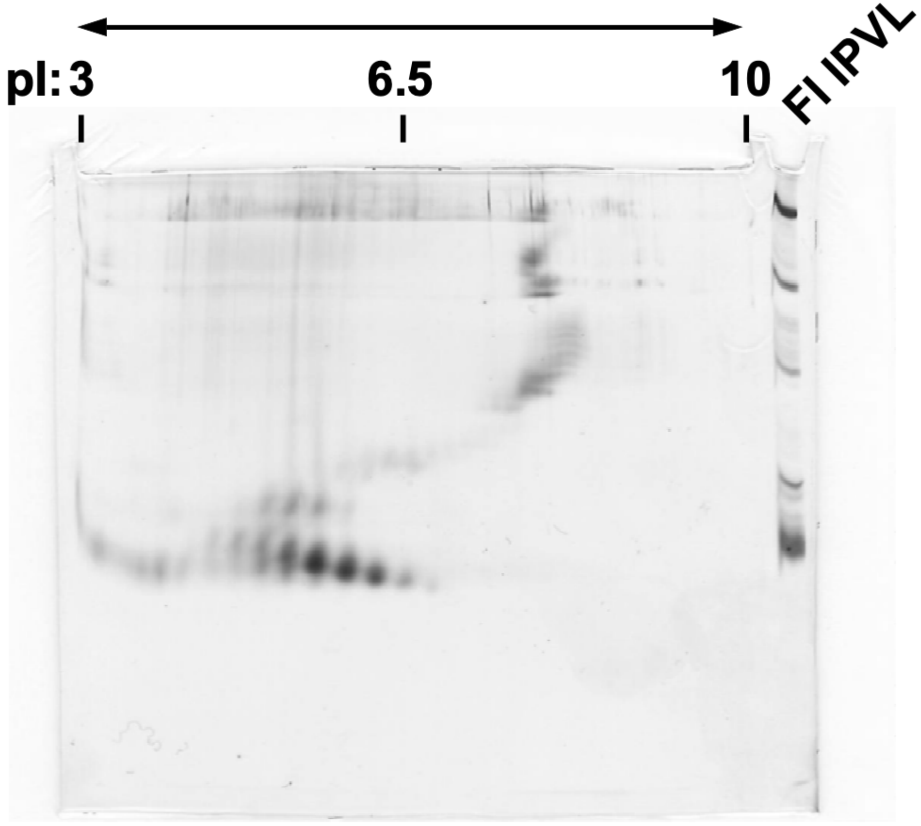

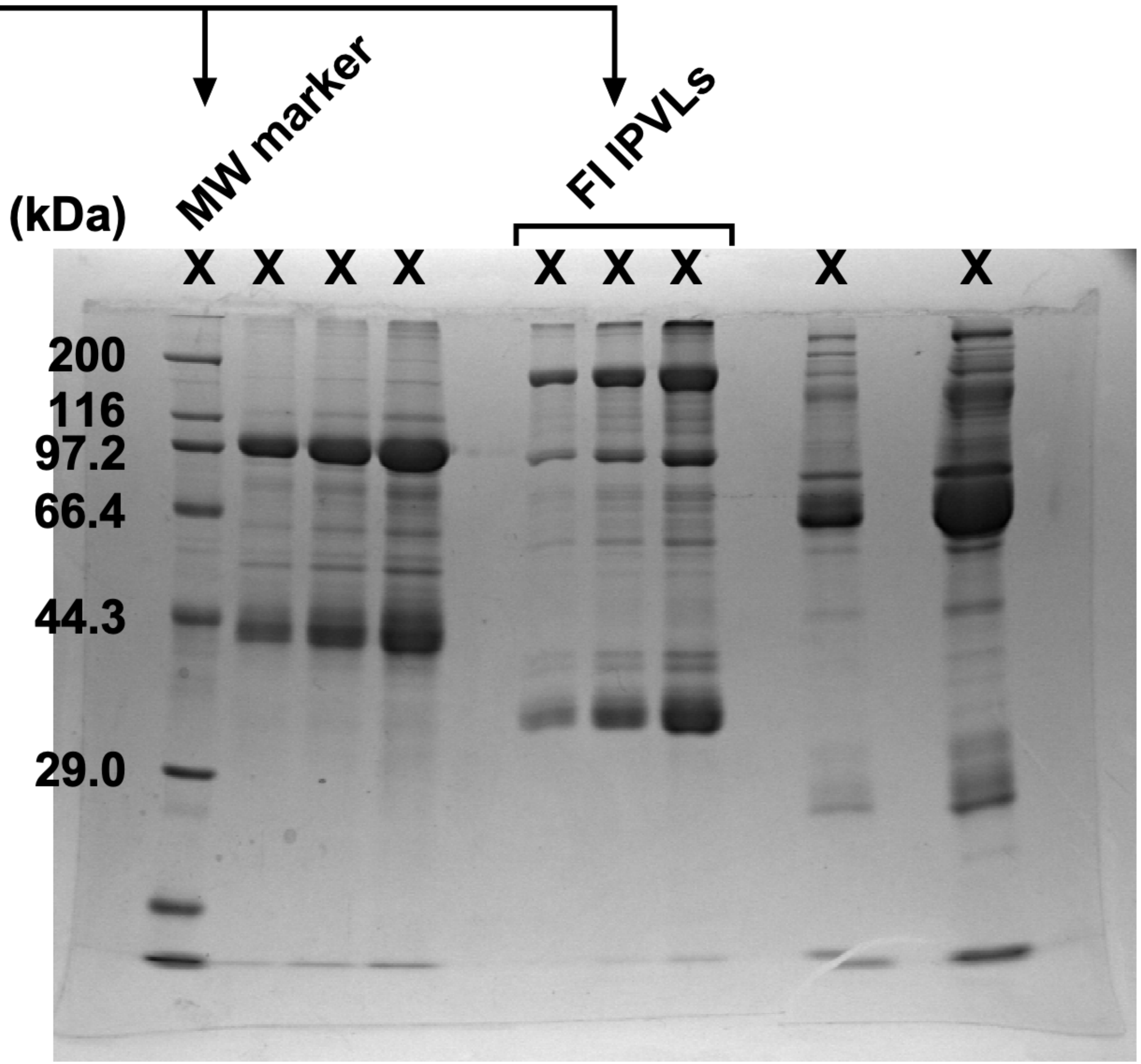

• F1 IPVL of the commercial White-Leghorn hen (88 μg wet weight) was subjected to 2D-PAGE under the non-reducing condition.

• Proteins in the 2D-gel were visualized by silver staining.

• Image of dried gel was obtained using LuminoGraph II (ATTO, Japan).

Original gel image for panel 2 of Fig3A and S3B Fig

• The pI labeling depended on pH range of the used gel strip.

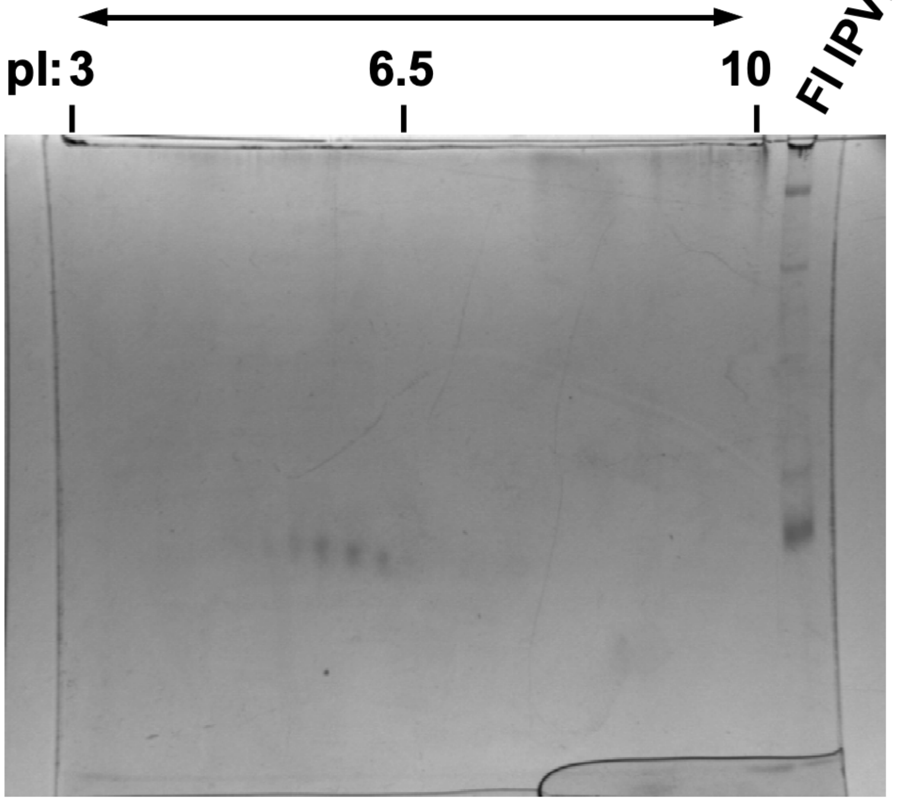

• F1 IPVL of the WL-G hen (180 μg wet weight) was subjected to 2D-PAGE under the non-reducing condition.

• Proteins in the 2D-gel were visualized by CBB staining.

• Image of dried gel was obtained using LuminoGraph II (ATTO, Japan).

Original blot image for panel 3 of Fig3A and S3C Fig

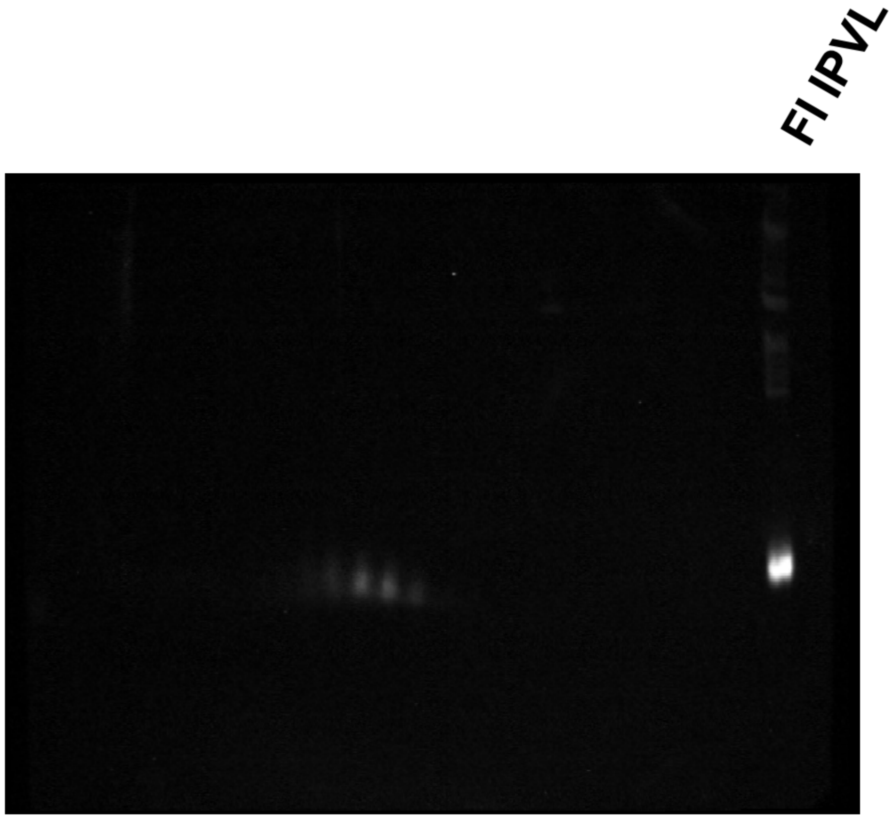

• F1 IPVL of the the commercial White-Leghorn hen (88 μg wet weight) was subjected to 2D-PAGE under the non-reducing condition.

• ZP3 isoforms in the 2D-gel were detected by Western blotting using the anti-ZP3 as a primary antibody.

• Image of dried gel was obtained using LuminoGraph II (ATTO, Japan).

Original blot image for panel 4 of Fig3A and S3D Fig

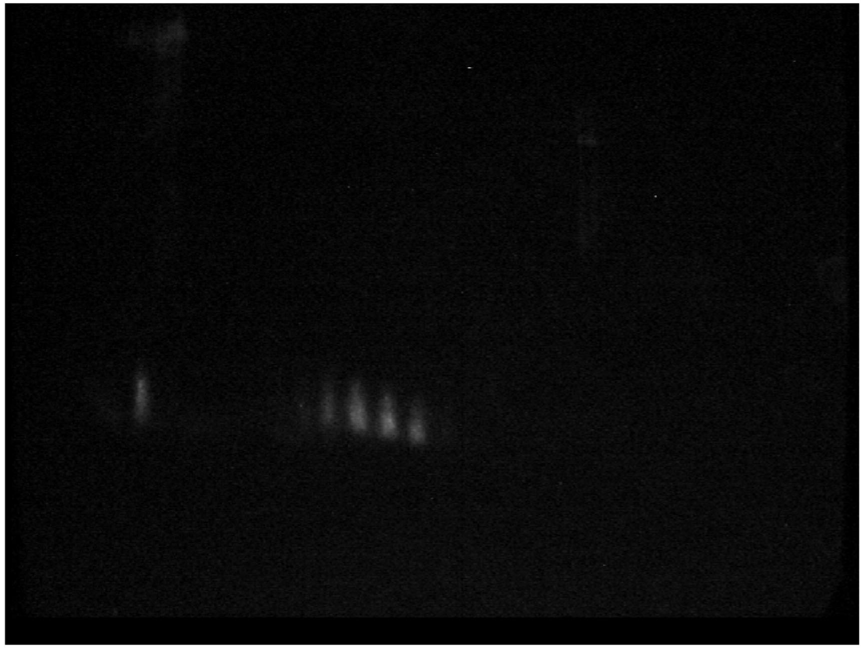

- F2 IPVL of the the commercial White-Leghorn hen (88 μg wet weight) was subjected to 2D-PAGE under the non-reducing condition.
- ZP3 isoforms in the 2D-gel were detected by Western blotting using the anti-ZP3 as a primary antibody.
- Image of dried gel was obtained using LuminoGraph II (ATTO, Japan).

Original blot image for panel 5 of Fig3A and S3E Fig

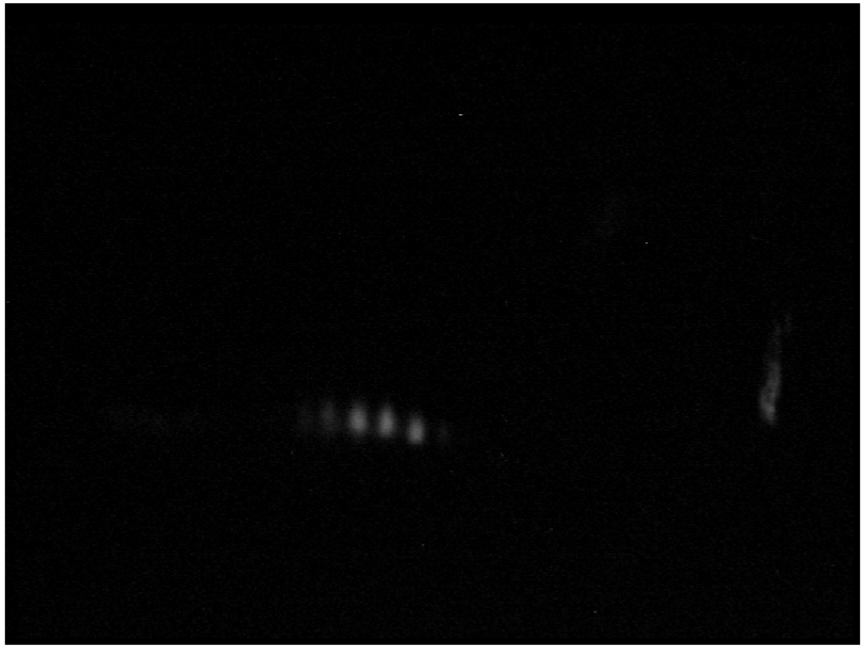

- F3 IPVL of the the commercial White-Leghorn hen (88 μg wet weight) was subjected to 2D-PAGE under the non-reducing condition.
- ZP3 isoforms in the 2D-gel were detected by Western blotting using the anti-ZP3 as a primary antibody.
- Image of dried gel was obtained using LuminoGraph II (ATTO, Japan).

Original gel image for Fig5 and S4 Fig (silver stain)

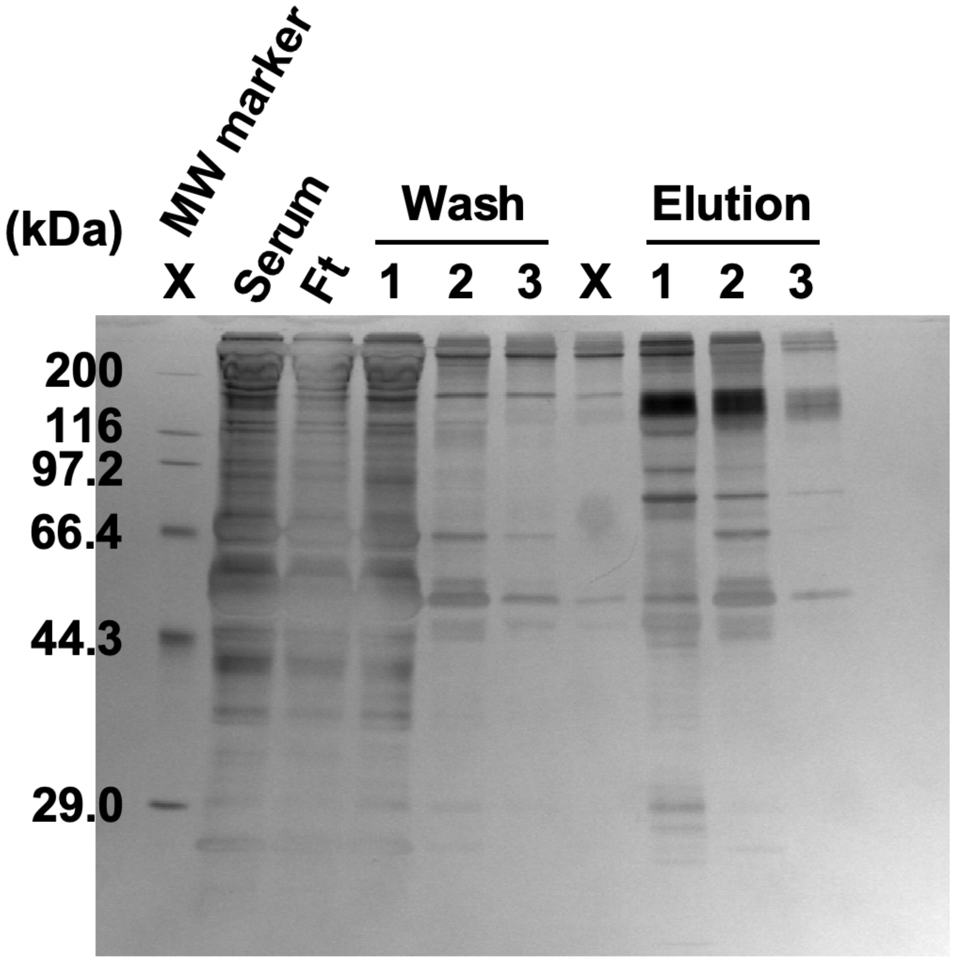

- Serum of the the commercial White-Leghorn hen (0.2 μl), flow-through (Ft; 0.4 μl) and each the wash and elution fractions (5.0 μl) were subjected to SDS-PAGE under the non-reducing conditions.
- Proteins in the gel were detected by silver staining.
- Image of gel was obtained using LuminoGraph II (ATTO, Japan).

Original blot image for Fig5 and S4 Fig (anti-ZP1)

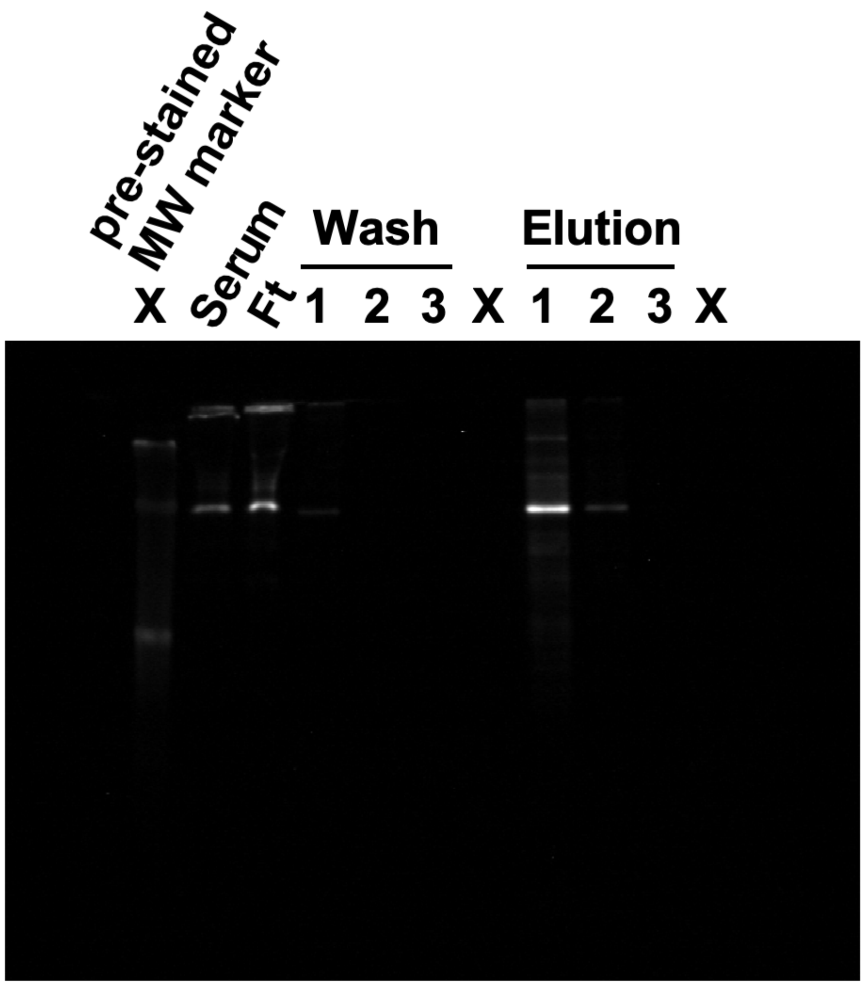

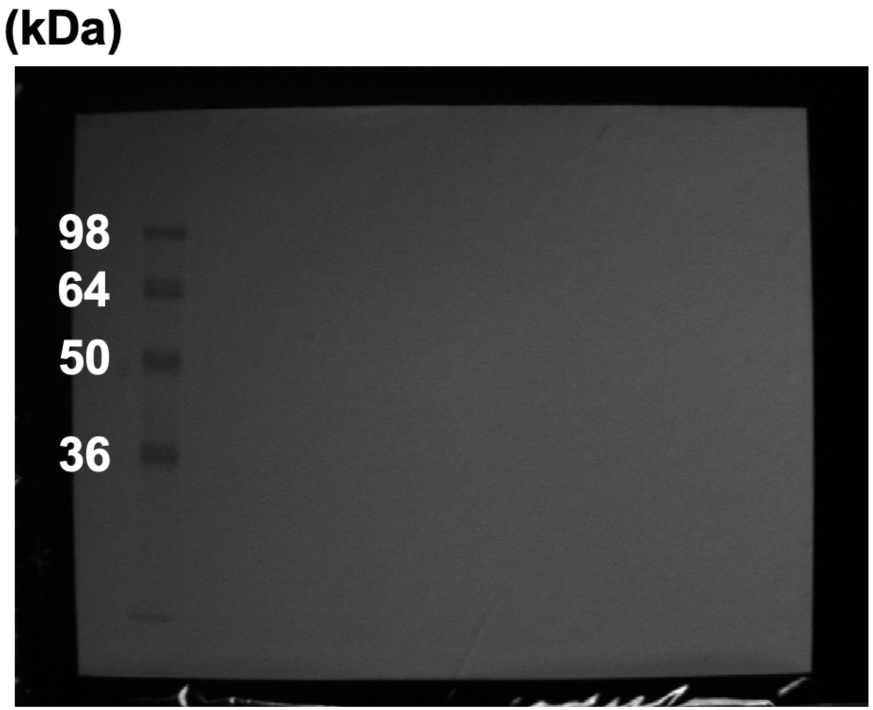

- Serum of the the commercial White-Leghorn hen (1.0 µl), flow-through (Ft; 2.0 µl) and each the wash and elution fractions (5.0 µl) were subjected to SDS-PAGE under the non-reducing conditions.
- ZP1 in the gel were detected by Western blotting using the anti-ZP1 repeat as a primary antibody.
- Image of blot was obtained using LuminoGraph II (ATTO, Japan).

- Light -field image of the membrane. Band-positions of the pre-stained MW marker were used to match the gel and blot images.

Original gel image for S5A Fig

• The MW labeling depended on the comparison of these lanes.

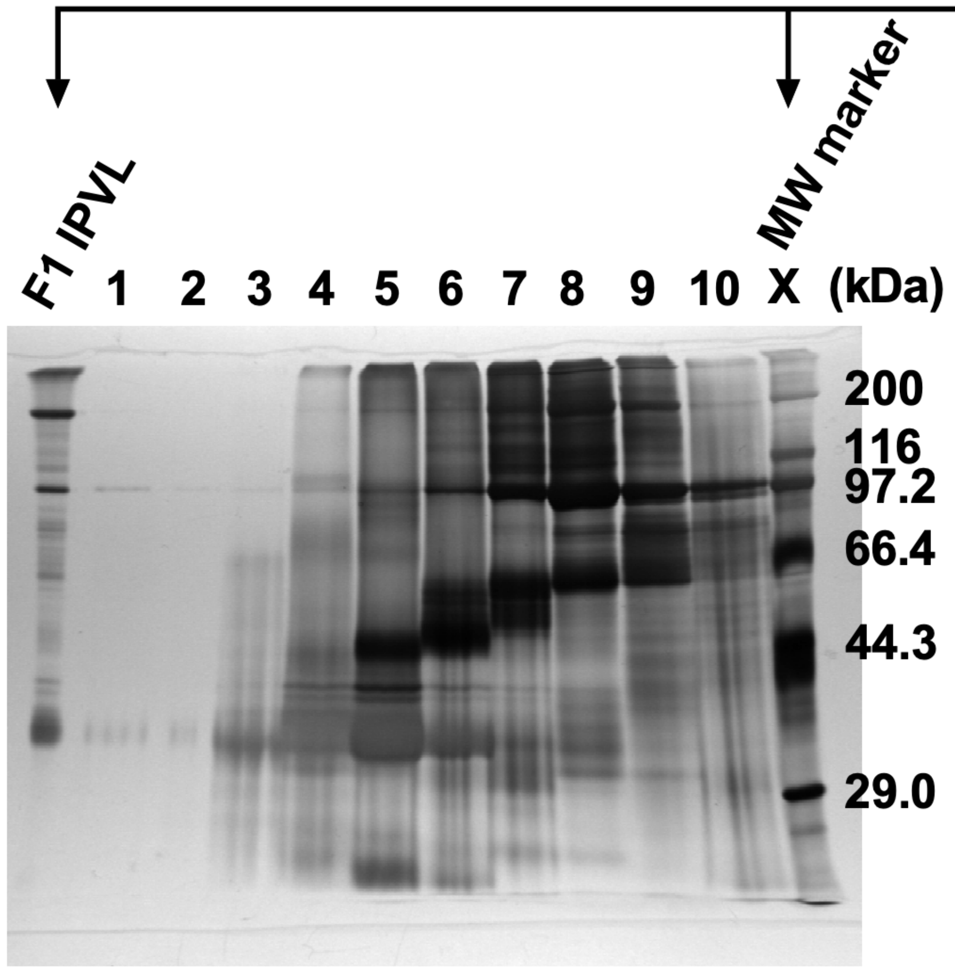

- F1 IPVL of the the commercial White-Leghorn hen (33 µg of wet weight) was subjected to the liquid-phase isoelectric focusing (LP-IEF) using carrer ampholyte for pH range of ~3.5–9.5, and the collected fractions 1 to 10 (each 5.0 µl) were subjected to SDS-PAGE under non-reducing conditions.
- Proteins in the gel were detected by silver stain.
- Image of blot was obtained using LuminoGraph II (ATTO, Japan).

Original gel image for Fig6A and S5B Fig

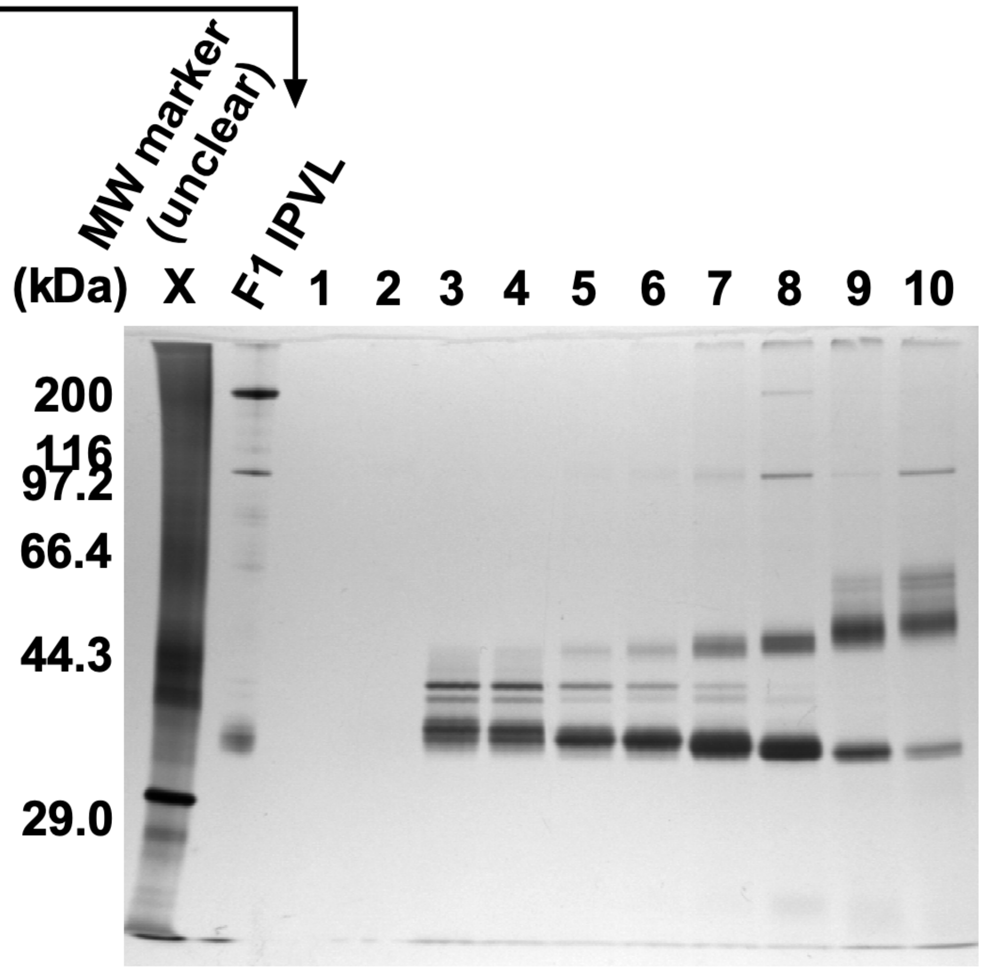

- Fractions 5 and 6 of the LP-IEF using carrer ampholyte for pH range of ~3.5–9.5 were pooled and re-fractionated. The collected fractions 1 to 10 (each 5.0 µl) of the re-fractionation were subjected to SDS-PAGE under non-reducing conditions.
- Proteins in the gel were detected by silver stain.
- Image of blot was obtained using LuminoGraph II (ATTO, Japan).

Reference gel image for Fig6B and original gel image for panels IPVL of S5C Fig (Mixture of IEF markers 1, 2 and F1 IPVL)

- The pI labeling depended on pH range of the used gel strip.

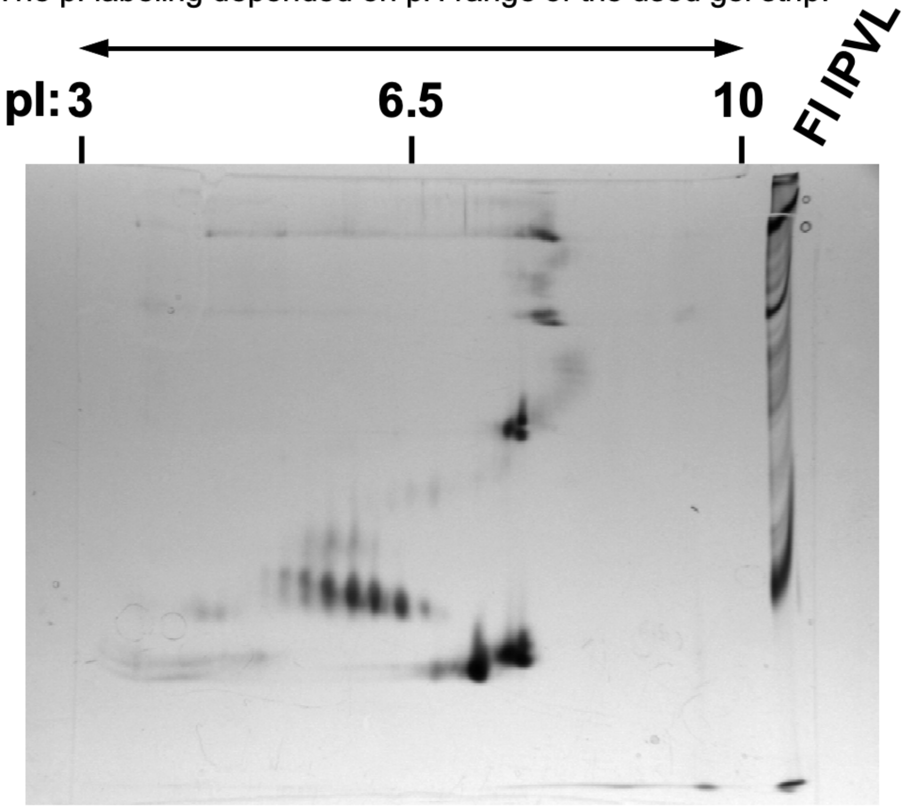

- Mixture of carbonic Anhydrase Isozyme I and II from human and bovine erythrocytes, respectively (each 0.2 μg; Merck, Germany) and F1 IPVL (88 μg wet weight) was subjected to 2D-PAGE under the non-reducing condition. This image was used as the standard for matching 2D-gel images.
- Proteins in the 2D-gel were visualized by silver staining.
- Image of dried gel was obtained using LuminoGraph II (ATTO, Japan).

Original gel image for panel a of Fig6B and panel 3(a) of S5C Fig

FI IPVL

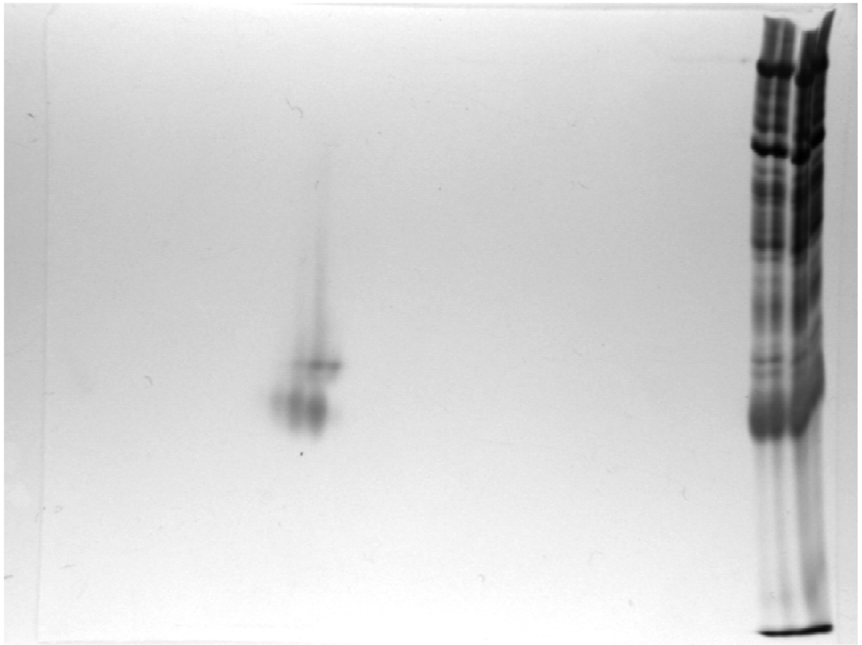

- An aliquote (10 µl) of dialyzed fraction 3 of the re-fractionation followed by the LP-IEF using carrier ampholyte for pH range of ~3.5–9.5 was subjected to 2D-PAGE under non-reducing condition.
- Proteins in the 2D-gel were visualized by silver staining.
- Image of dried gel was obtained using LuminoGraph II (ATTO, Japan).

Original gel image for panel 4 of S5C Fig

FI IPVL (failed to be stained)

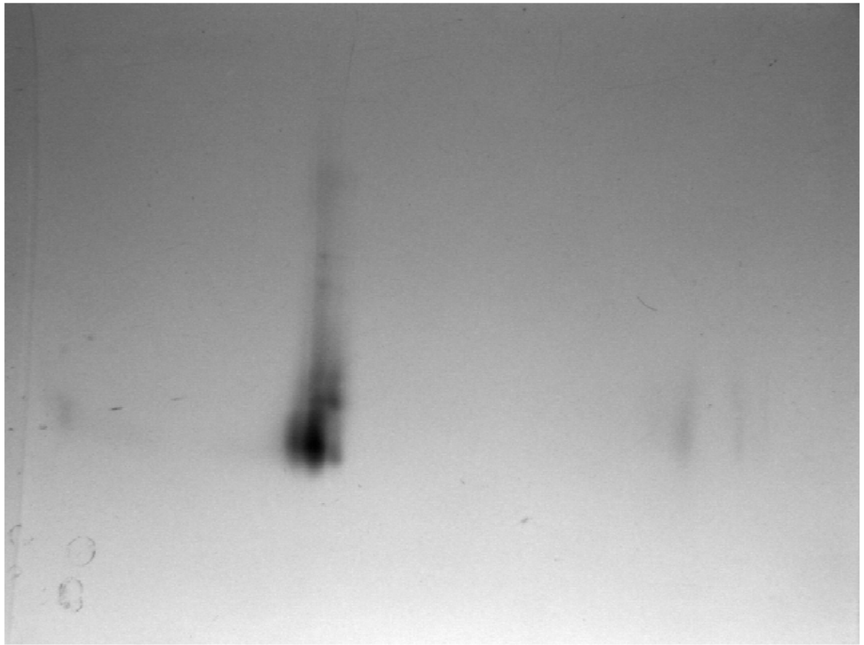

- An aliquote (10 µl) of dialyzed fraction 4 of the re-fractionation followed by the LP-IEF using carrier ampholyte for pH range of ~3.5–9.5 was subjected to 2D-PAGE under non-reducing condition.
- Proteins in the 2D-gel were visualized by silver staining.
- Image of dried gel was obtained using LuminoGraph II (ATTO, Japan).

Original gel image for panel 5 of S5C Fig

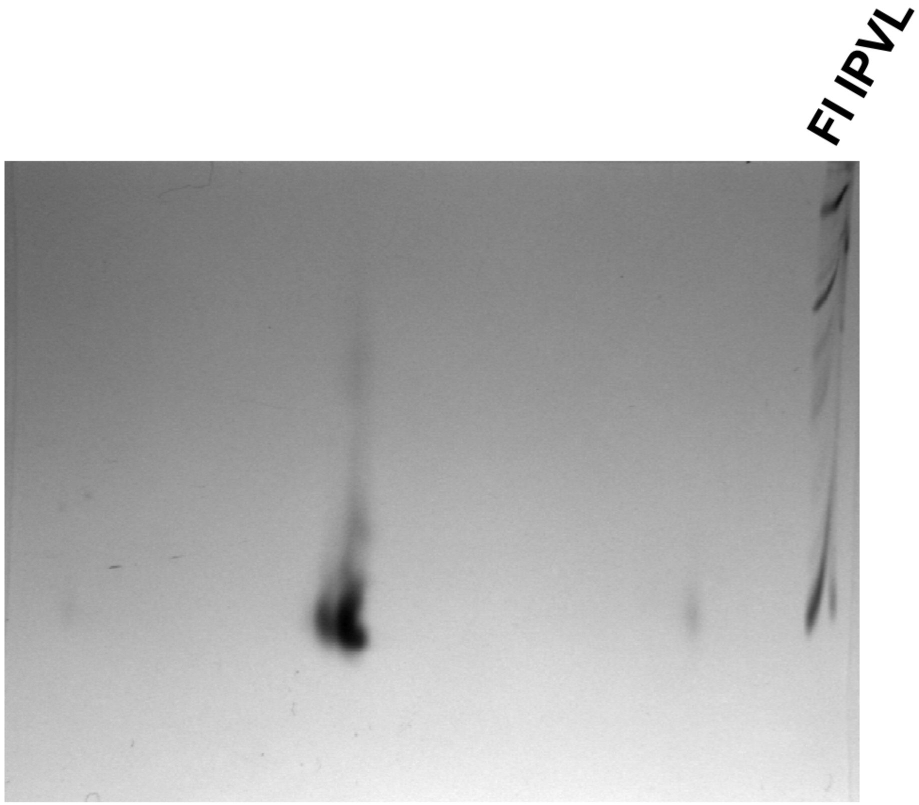

- An aliquote (10 µl) of dialyzed fraction 5 of the re-fractionation followed by the LP-IEF using carrier ampholyte for pH range of ~3.5–9.5 was subjected to 2D-PAGE under non-reducing condition.
- Proteins in the 2D-gel were visualized by silver staining.
- Image of dried gel was obtained using LuminoGraph II (ATTO, Japan).

Original gel image for panel 6 of S5C Fig

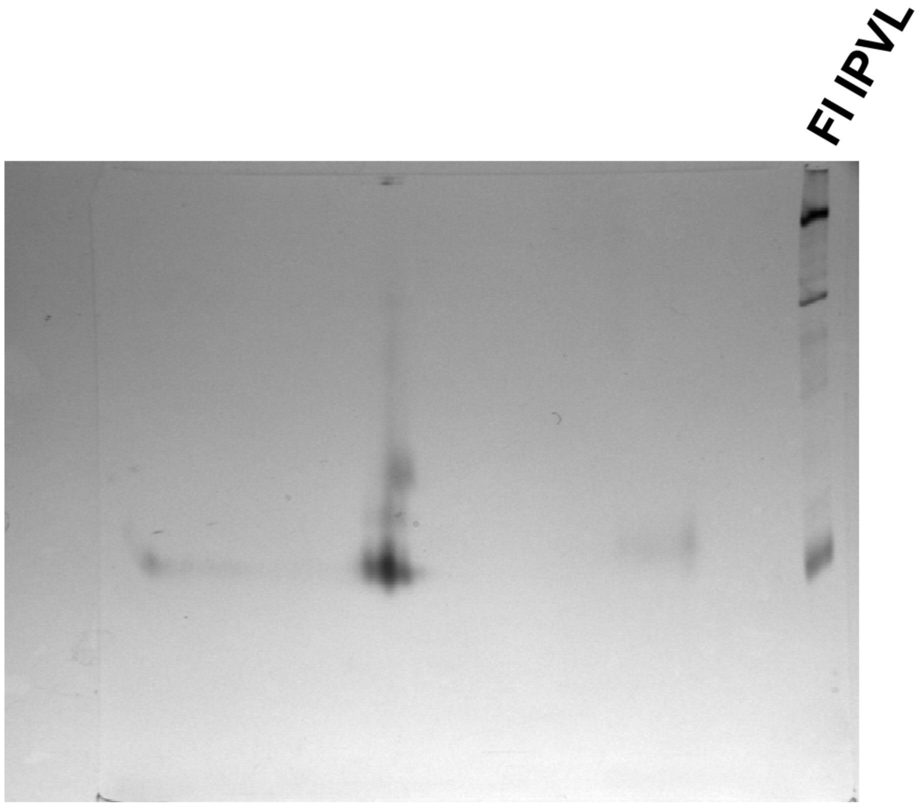

- An aliquote (10 µl) of dialyzed fraction 6 of the re-fractionation followed by the LP-IEF using carrier ampholyte for pH range of ~3.5–9.5 was subjected to 2D-PAGE under non-reducing condition.
- Proteins in the 2D-gel were visualized by silver staining.
- Image of dried gel was obtained using LuminoGraph II (ATTO, Japan).

Original gel image for panel b of Fig6B and panel 7(b) of S5C Fig

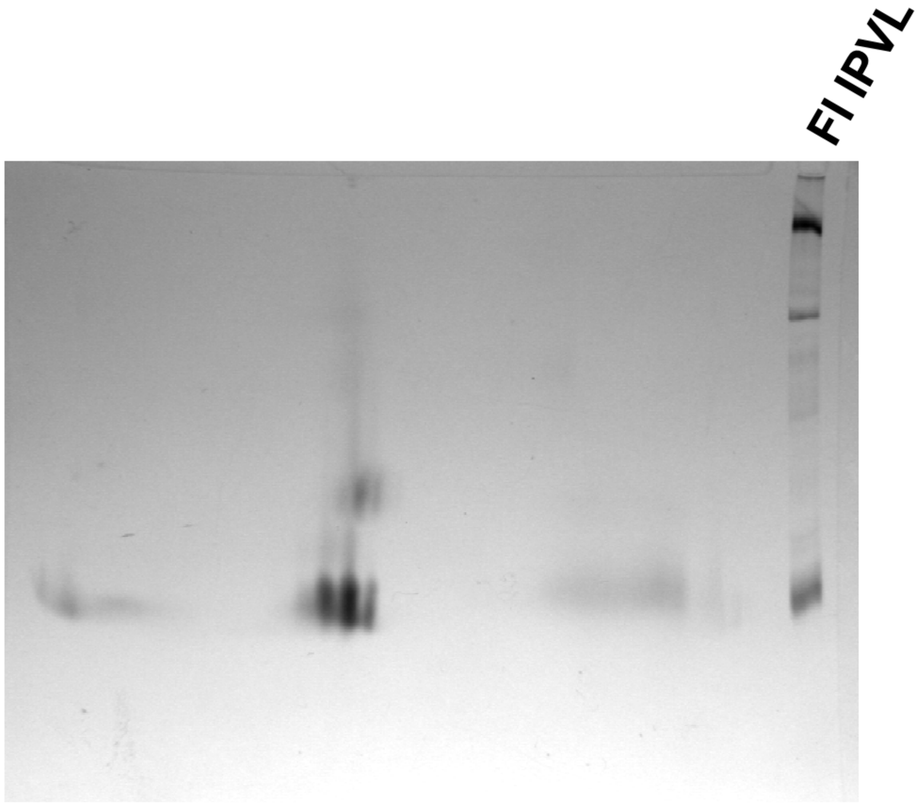

- An aliquote (10 µl) of dialyzed fraction 7 of the re-fractionation followed by the LP-IEF using carrier ampholyte for pH range of ~3.5–9.5 was subjected to 2D-PAGE under non-reducing condition.
- Proteins in the 2D-gel were visualized by silver staining.
- Image of dried gel was obtained using LuminoGraph II (ATTO, Japan).

Original gel image for panel 8 of S5C Fig

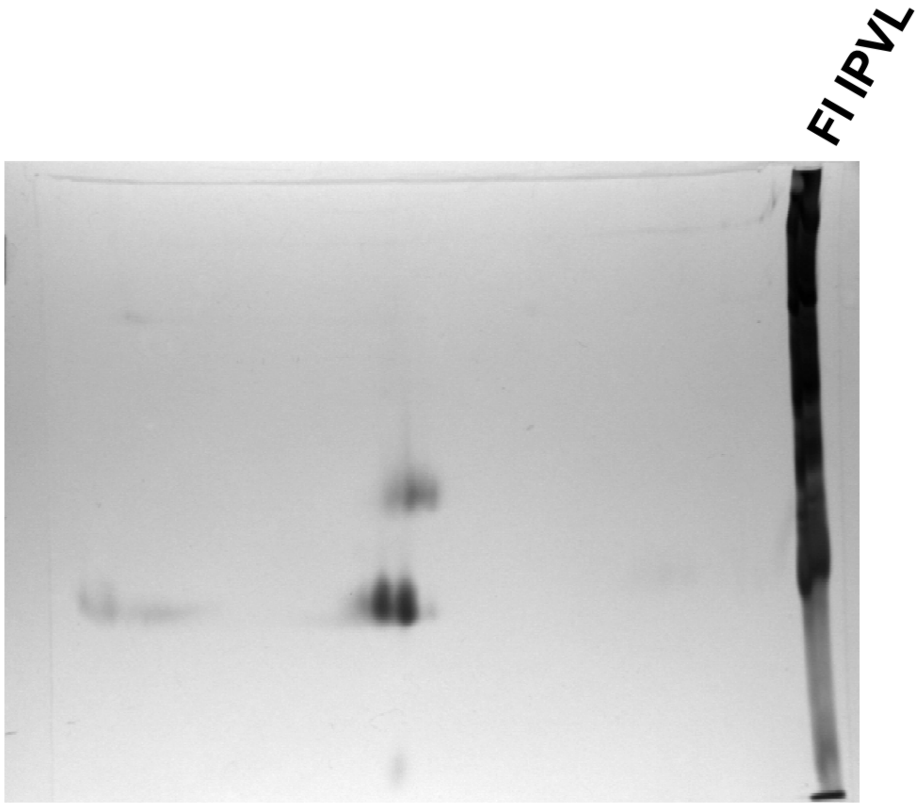

- An aliquote (10 µl) of dialyzed fraction 8 of the re-fractionation followed by the LP-IEF using carrier ampholyte for pH range of ~3.5–9.5 was subjected to 2D-PAGE under non-reducing condition.
- Proteins in the 2D-gel were visualized by silver staining.
- Image of dried gel was obtained using LuminoGraph II (ATTO, Japan).

Original gel image for panel 9 of S5C Fig

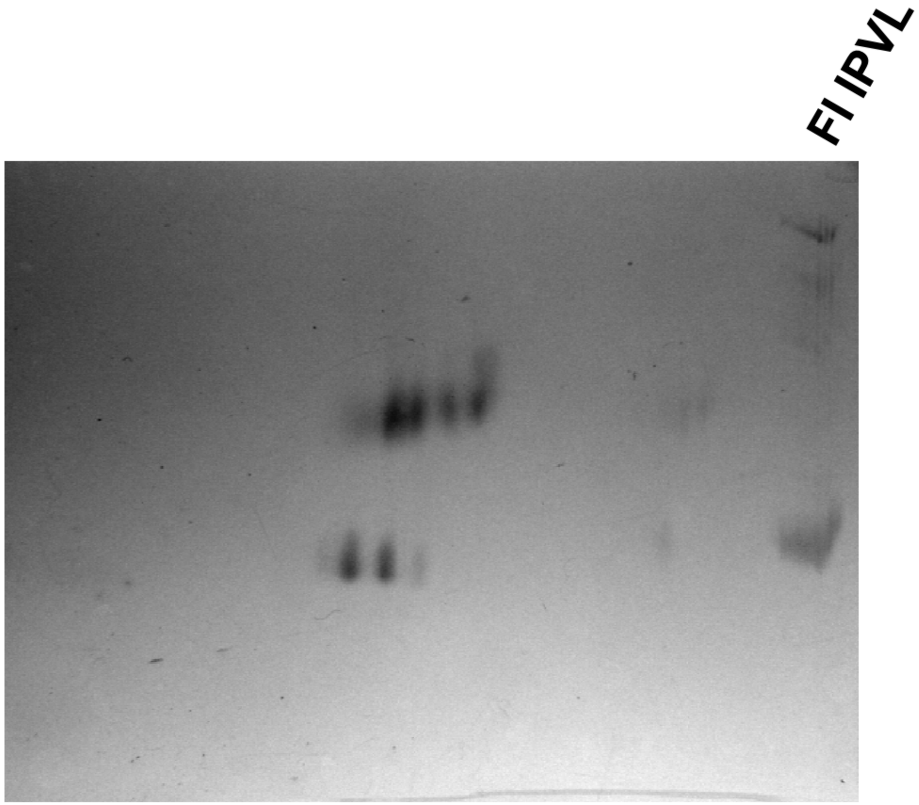

• An aliquote (10 μl) of dialyzed fraction 9 of the re-fractionation followed by the LP-IEF using carrier ampholyte for pH range of ~3.5–9.5 was subjected to 2D-PAGE under non-reducing condition.

• Proteins in the 2D-gel were visualized by silver staining.

• Image of dried gel was obtained using LuminoGraph II (ATTO, Japan).

Original gel image for panel c of Fig6B and panel 10(c) of S5C Fig

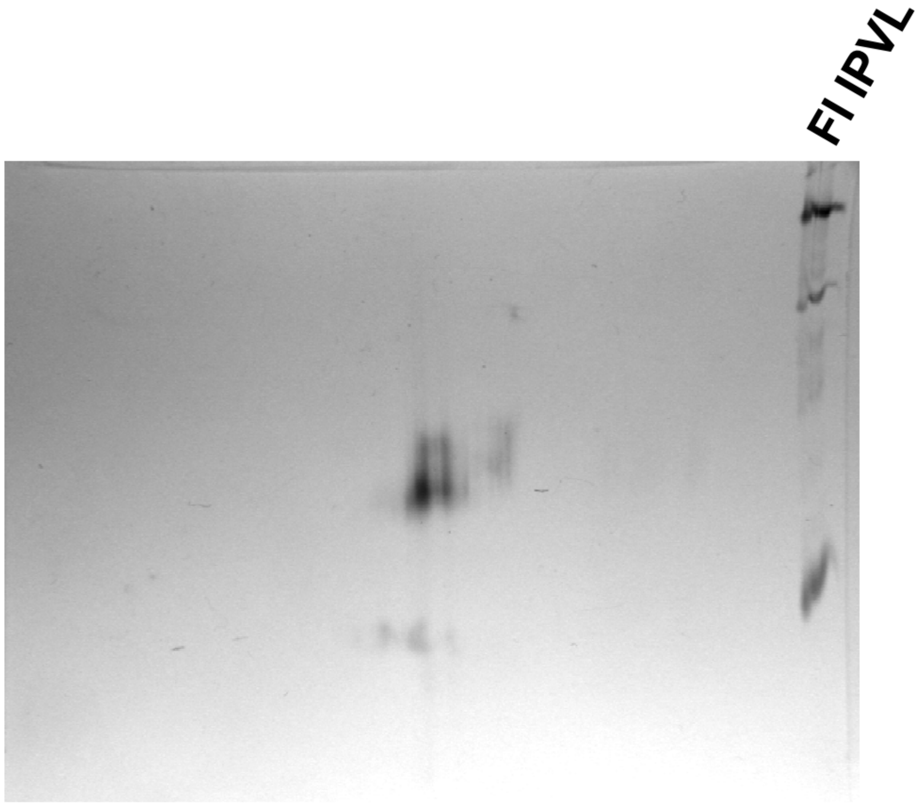

• An aliquote (10 μl) of dialyzed fraction 10 of the re-fractionation followed by the LP-IEF using carrier ampholyte for pH range of ~3.5–9.5 was subjected to 2D-PAGE under non-reducing condition.

• Proteins in the 2D-gel were visualized by silver staining.

• Image of dried gel was obtained using LuminoGraph II (ATTO, Japan).

Original gel image for
S5D Fig

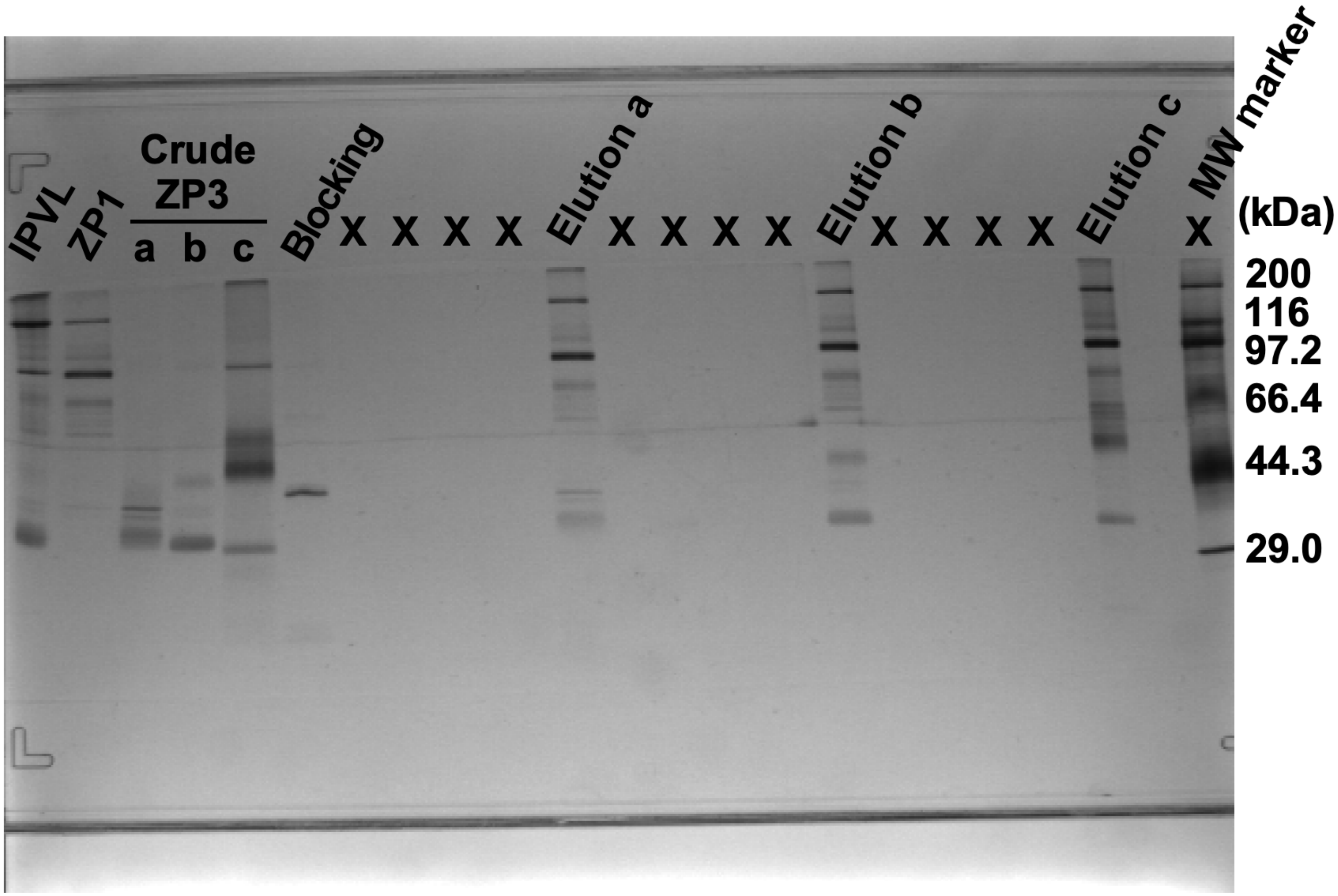

Original blot image for Fig6C and S6E Fig (anti-ZP3)

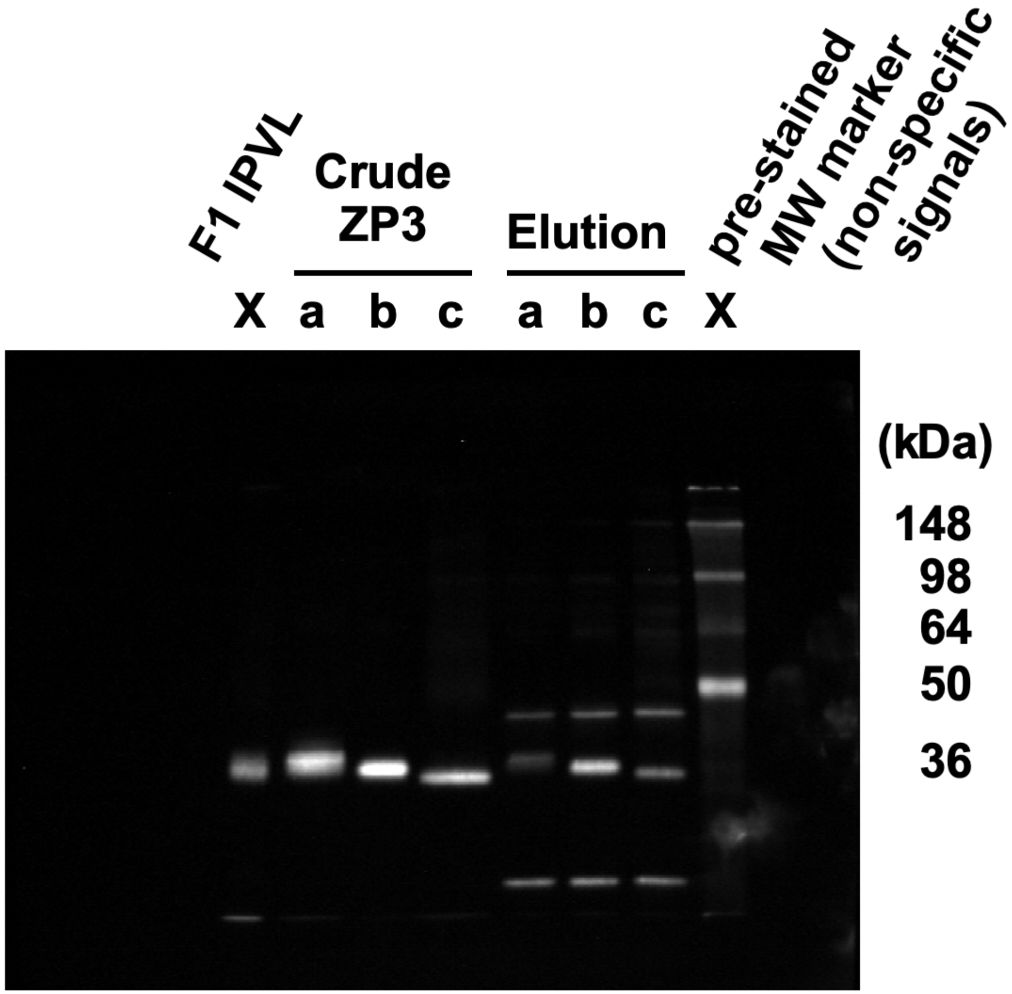

- The Crude ZP3-isoform fractions a–c and the corresponding elution fractions a–c were subjected to SDS-PAGE under non-reducing conditions.
- ZP3 in the gel were detected by Western blotting using the anti-ZP3 as a primary antibody.
- Image of blot was obtained using LuminoGraph II (ATTO, Japan).

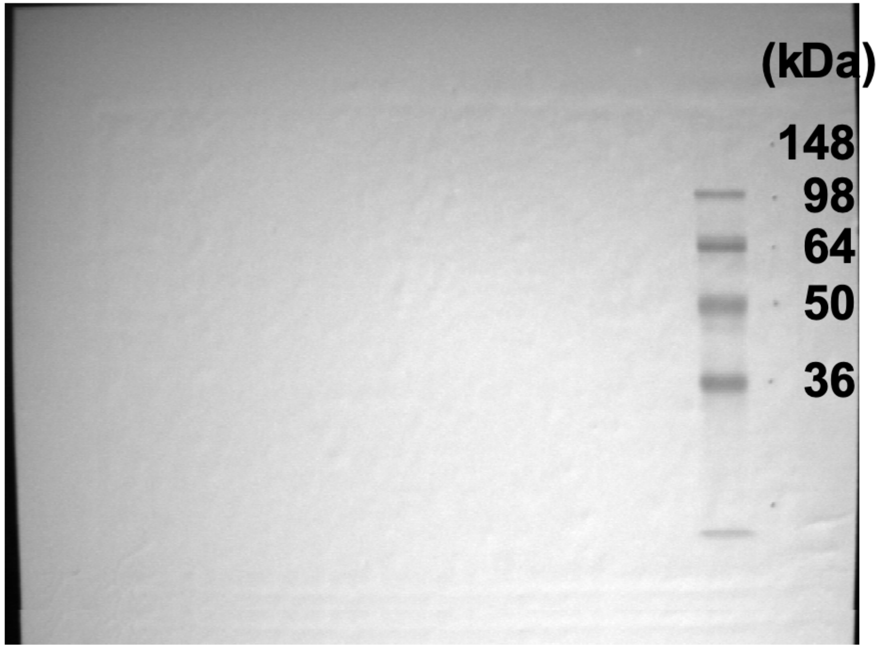

- Light -field image of the membrane.
  Band-positions of the pre-stained MW marker were labeled.
  Some of the markers were non-specifically detrected by Western blotting.

Original blot image for
Fig6C and S6E Fig (re-probed by anti-ZP1)

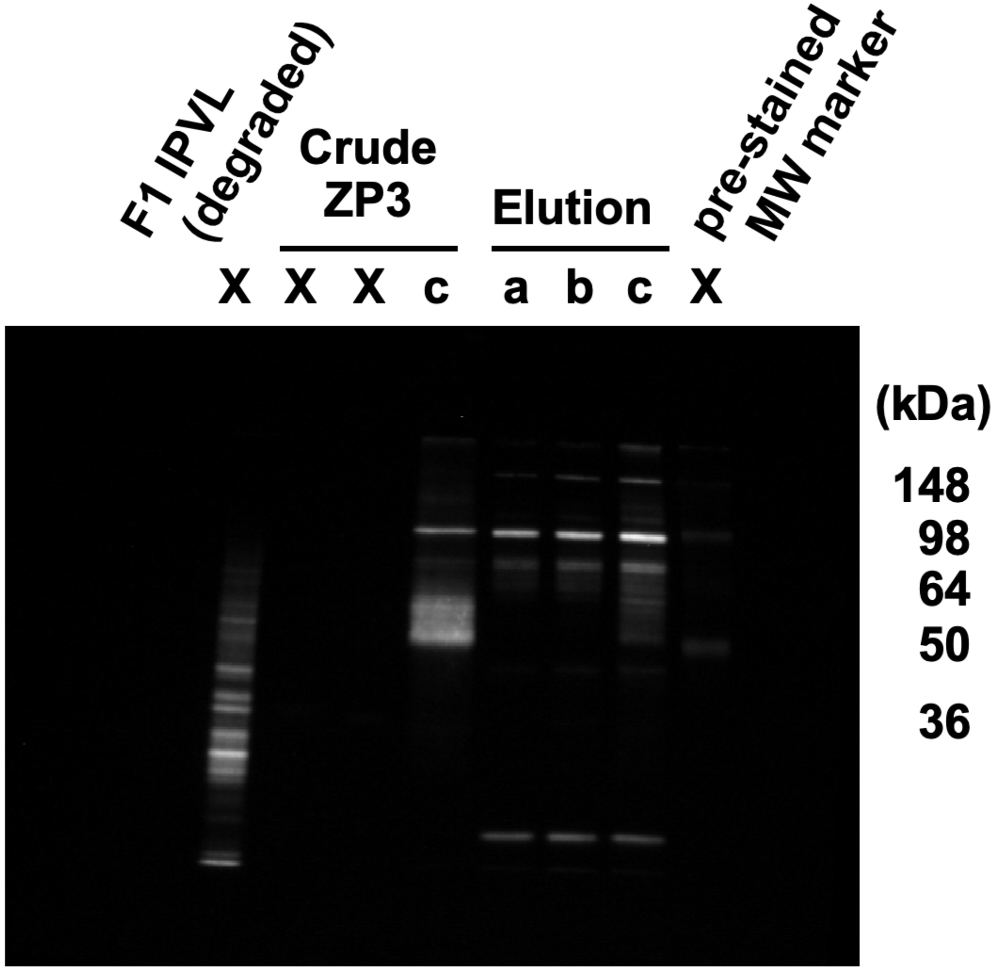

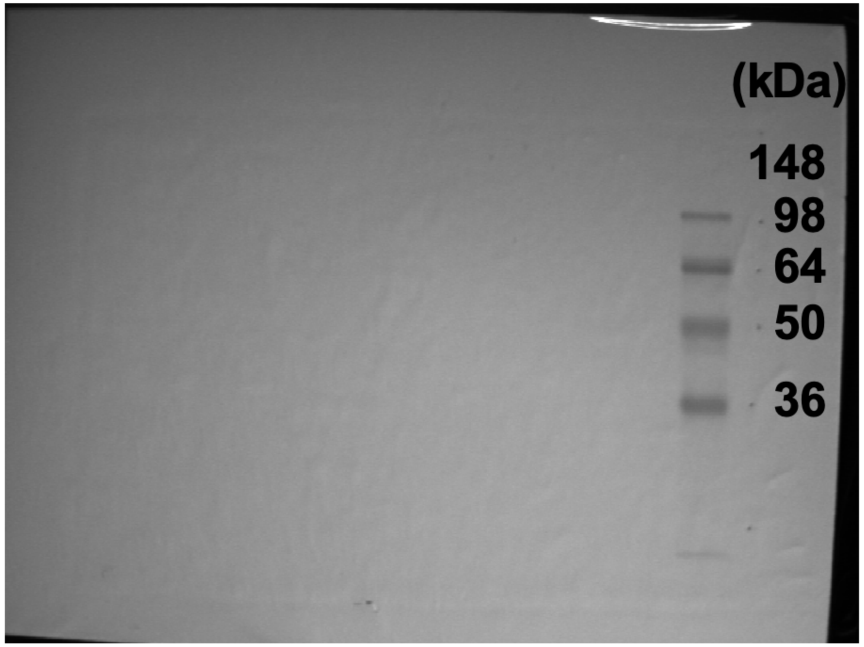

- The Crude ZP3-isoform fractions a–c and the corresponding elution fractions a–c were subjected to SDS-PAGE under non-reducing conditions.
- ZP1 in the gel were detected by re-probing of the membrane using the anti-ZP1 repeat as a primary antibody.
- Image of blot was obtained using LuminoGraph II (ATTO, Japan).

- Light -field image of the membrane.
  Band-positions of the pre-stained MW marker were labeled.

Original gel image for S5F Fig

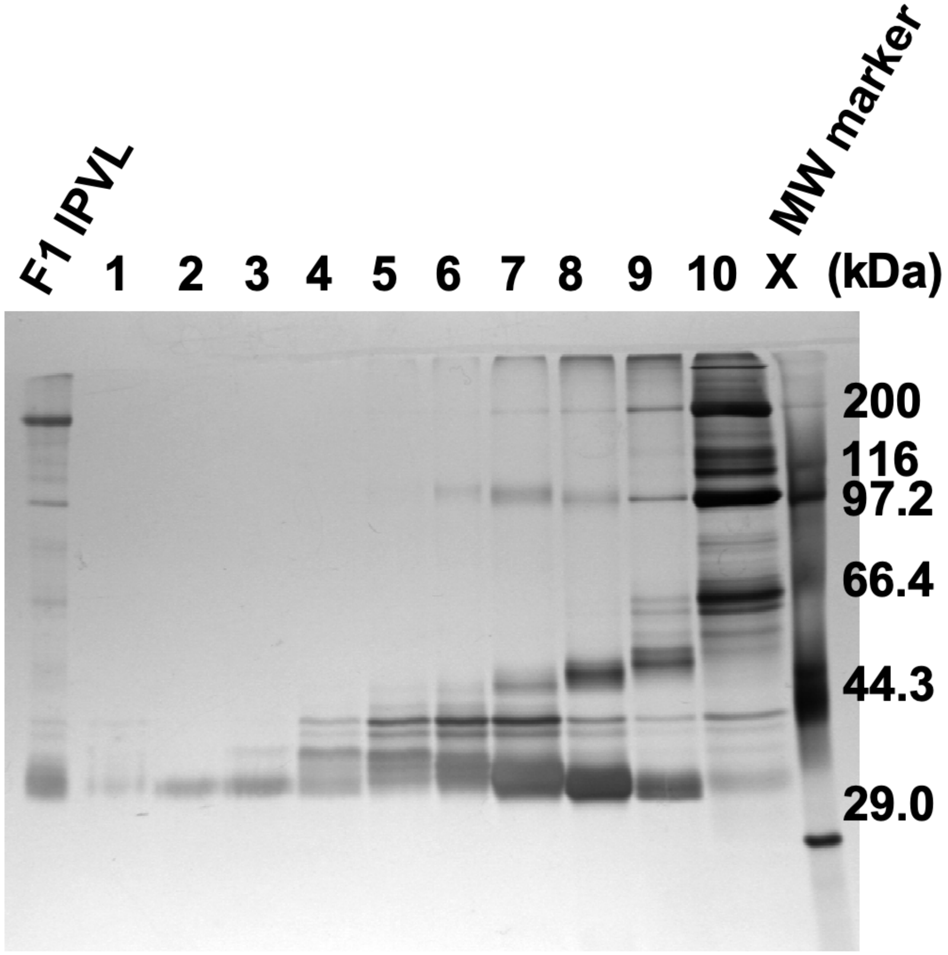

- F1 IPVL of the the commercial White-Leghorn hen (29 µg of wet weight) was subjected to the liquid-phase isoelectric focusing (LP-IEF) using carrer ampholyte for pH range of ~5–7, and the collected fractions 1 to 10 (each 5.0 µl) were subjected to SDS-PAGE under non-reducing conditions.
- Proteins in the gel were detected by silver stain.
- Image of blot was obtained using LuminoGraph II (ATTO, Japan).

Original gel image for Fig6E and S5G Fig

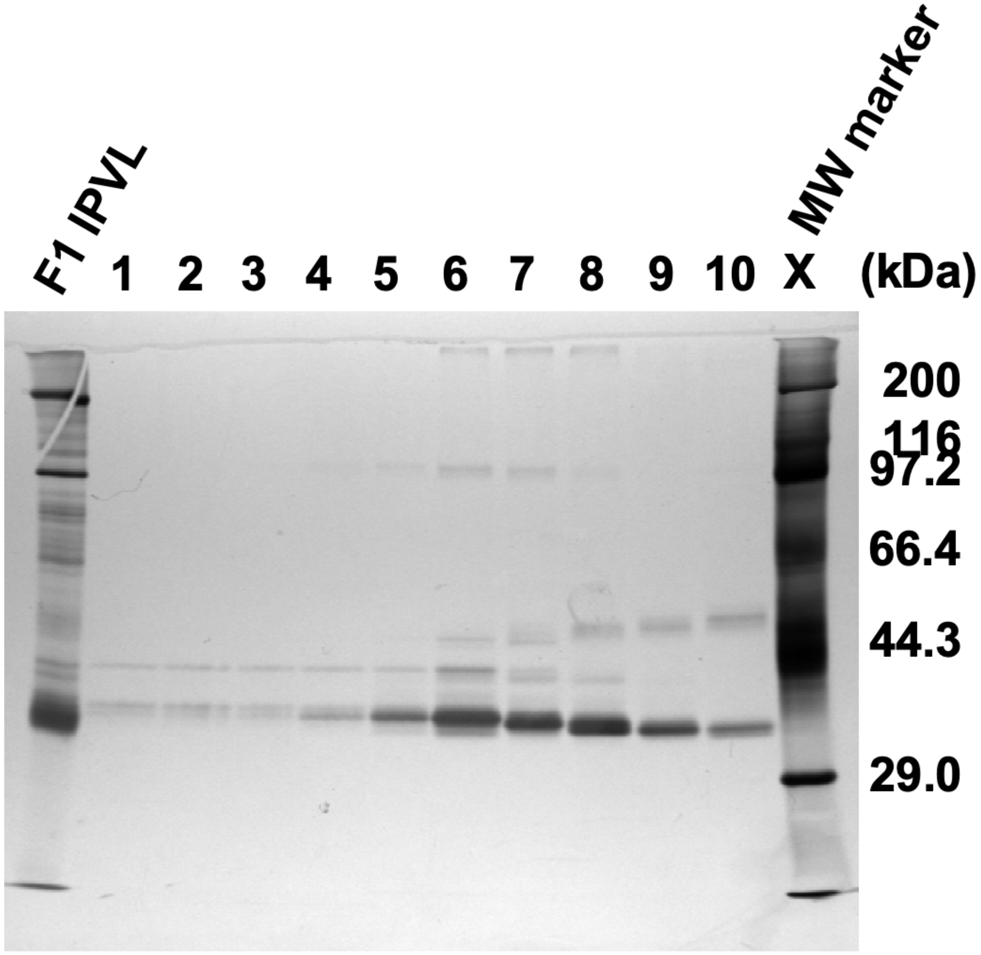

- Fractions 7–9 of the LP-IEF using carrer ampholyte for pH range of ~5–7 were pooled and re-fractionated. The collected fractions 1 to 10 (each 5.0 µl) of the re-fractionation were subjected to SDS-PAGE under non-reducing conditions.
- Proteins in the gel were detected by silver stain.
- Image of blot was obtained using LuminoGraph II (ATTO, Japan).

Reference gel image for Fig6F and S5H Fig (IEF marker 1)

• The pI labeling depended on pH range of the used gel strip.

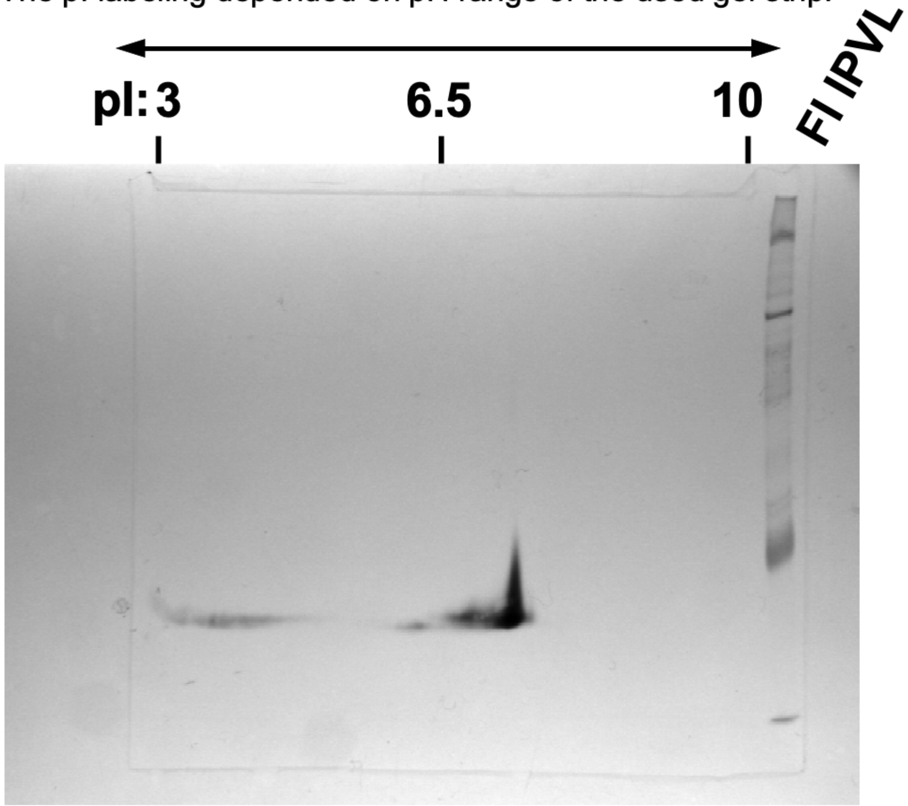

• Carbonic Anhydrase Isozyme II from bovine erythrocytes (0.2 µg; Merck, Germany) was subjected to 2D-PAGE under the non-reducing condition. This marker was used for internal controls for matching 2D-gel images.

• Proteins in the 2D-gel were visualized by silver staining.

• Image of dried gel was obtained using LuminoGraph II (ATTO, Japan).

Reference gel image for Fig6F and S5H Fig (IEF marker 2)

• The pI labeling depended on pH range of the used gel strip.

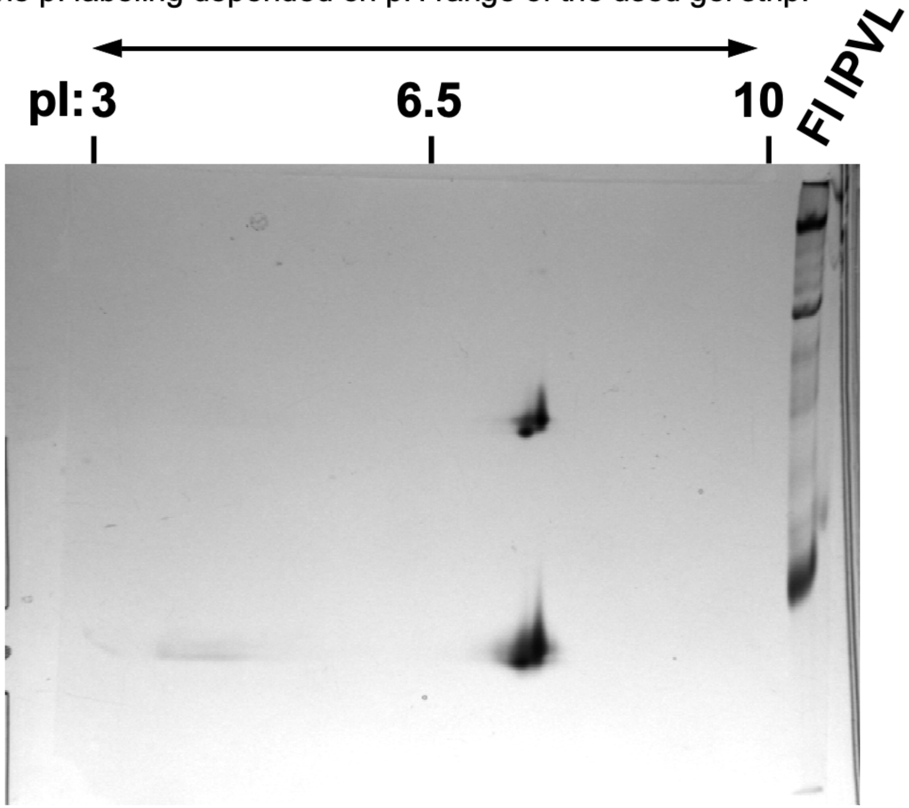

• Carbonic Anhydrase Isozyme I from human erythrocytes (0.2 µg; Merck, Germany) was subjected to 2D-PAGE under the non-reducing condition. This marker was used for internal controls for matching 2D-gel images.

• Proteins in the 2D-gel were visualized by silver staining.

• Image of dried gel was obtained using LuminoGraph II (ATTO, Japan).

Reference gel image for Fig6F and S5H Fig (Mixture of IEF markers 1 and 2)

• The pI labeling depended on pH range of the used gel strip.

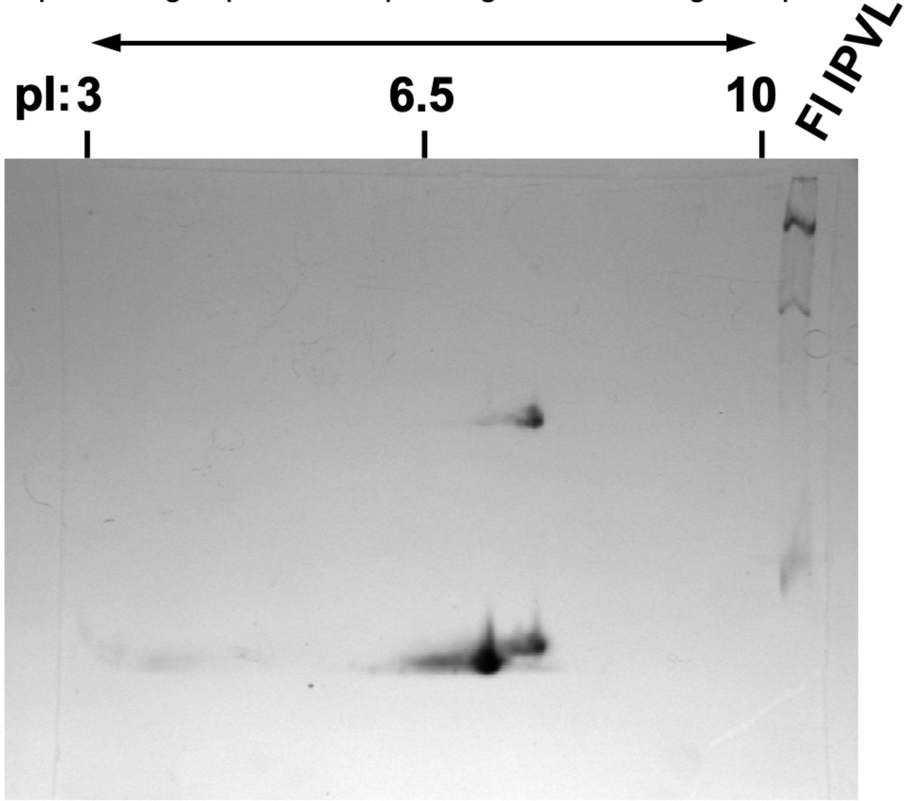

• Mixture of carbonic Anhydrase Isozyme I and II from human and bovine erythrocytes, respectively (each 0.2 μg; Merck, Germany) was subjected to 2D-PAGE under the non-reducing condition. These markers were used as internal controls for matching 2D-gel images.

• Proteins in the 2D-gel were visualized by silver staining.

• Image of dried gel was obtained using LuminoGraph II (ATTO, Japan).

Reference gel image for Fig6F and original gel image for panels IPVL of S5H Fig (Mixture of IEF markers 1, 2 and F1 IPVL)

• The pI labeling depended on pH range of the used gel strip.

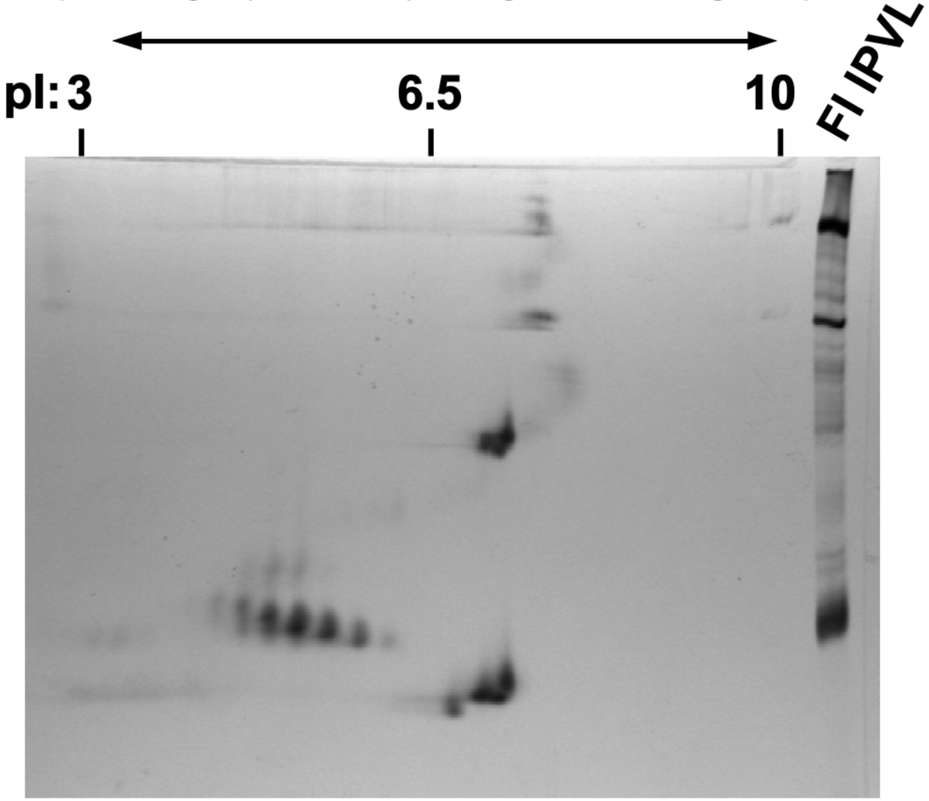

• Mixture of carbonic Anhydrase Isozyme I and II from human and bovine erythrocytes, respectively (each 0.2 μg; Merck, Germany) and F1 IPVL (88 μg wet weight) was subjected to 2D-PAGE under the non-reducing condition. This image was used as the standard for matching 2D-gel images.

• Proteins in the 2D-gel were visualized by silver staining.

• Image of dried gel was obtained using LuminoGraph II (ATTO, Japan).

Original gel image for panel 5 of S5H Fig (with IEF markers)

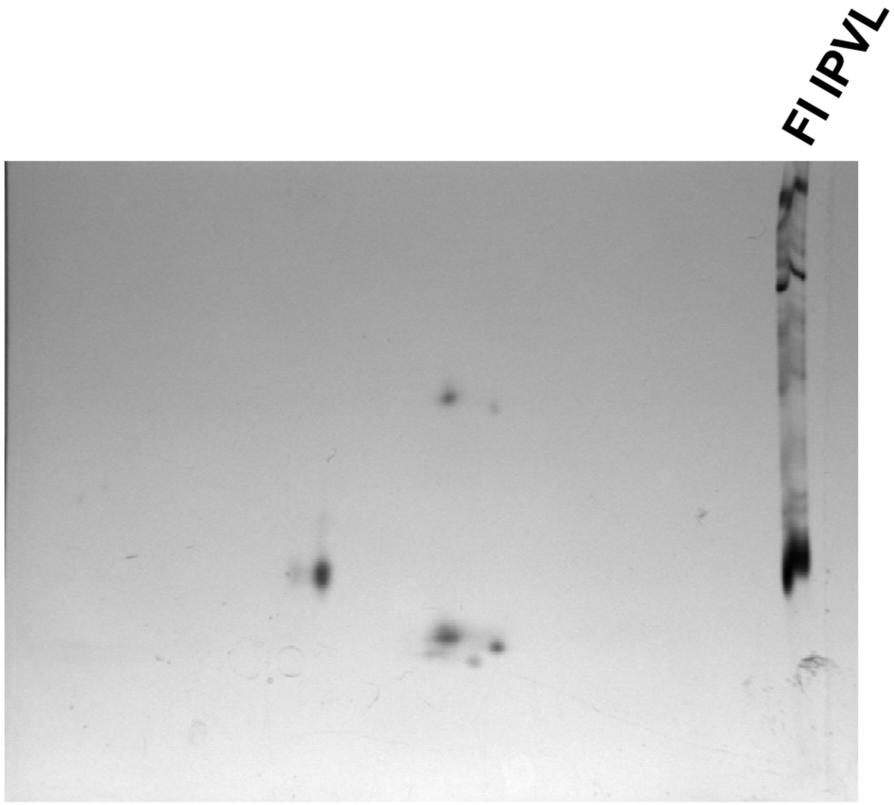

- An aliquote (10 μl) of dialyzed fraction 5 of the re-fractionation followed by the LP-IEF using carrier ampholyte for pH range of ~5–7 was subjected with the IEF markers to 2D-PAGE under non-reducing condition.
- Proteins in the 2D-gel were visualized by silver staining.
- Image of dried gel was obtained using LuminoGraph II (ATTO, Japan).

Original gel image for panel d of Fig6F and panel 6(d) of S5H Fig (with IEF markers)

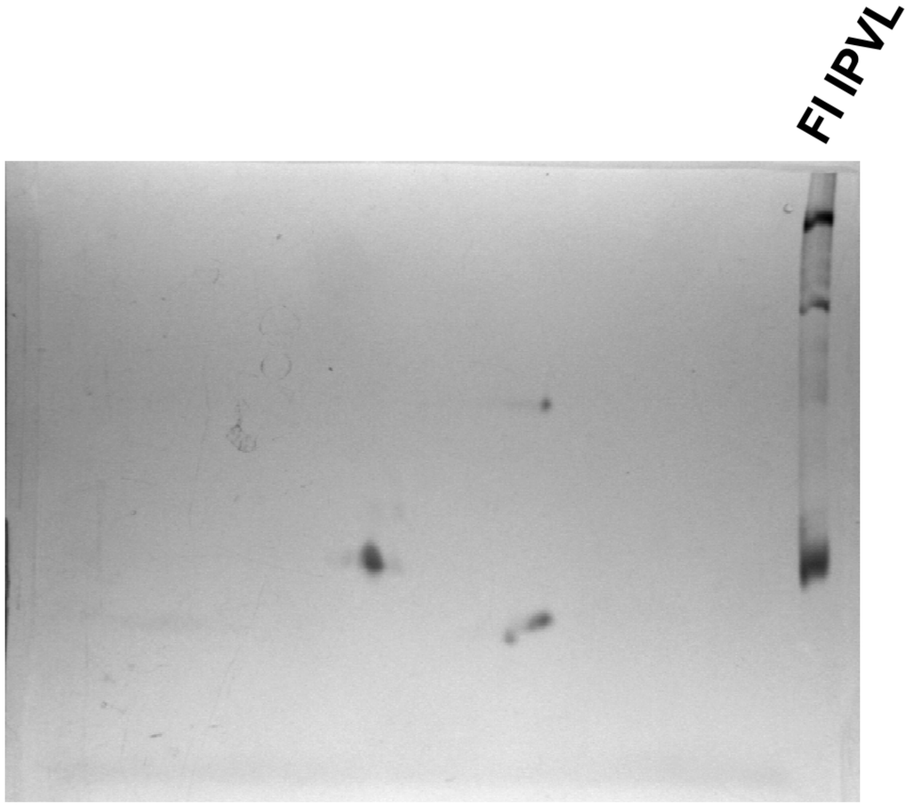

- An aliquote (10 μl) of dialyzed fraction 6 of the re-fractionation followed by the LP-IEF using carrier ampholyte for pH range of ~5–7 was subjected with the IEF markers to 2D-PAGE under non-reducing condition.
- Proteins in the 2D-gel were visualized by silver staining.
- Image of dried gel was obtained using LuminoGraph II (ATTO, Japan).

Original gel image for panel 7 of S5H Fig (with IEF markers)

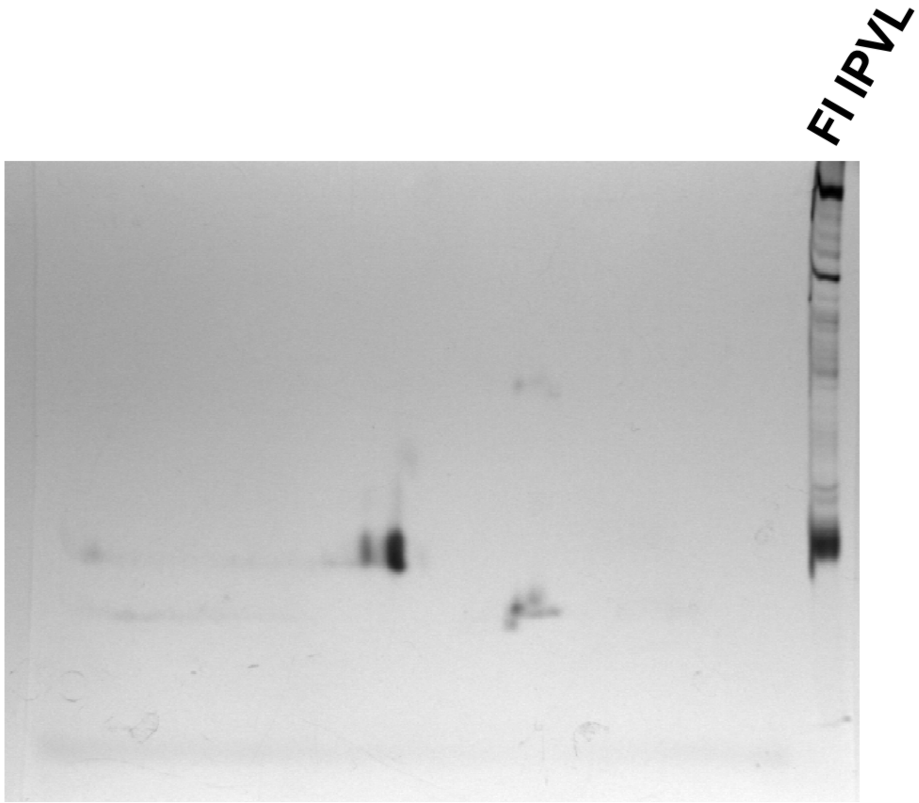

- An aliquote (10 μl) of dialyzed fraction 7 of the re-fractionation followed by the LP-IEF using carrier ampholyte for pH range of ~5–7 was subjected with the IEF markers to 2D-PAGE under non-reducing condition.
- Proteins in the 2D-gel were visualized by silver staining.
- Image of dried gel was obtained using LuminoGraph II (ATTO, Japan).

Original gel image for panel e of Fig6F and panel 8(a) of S5H Fig (with IEF markers)

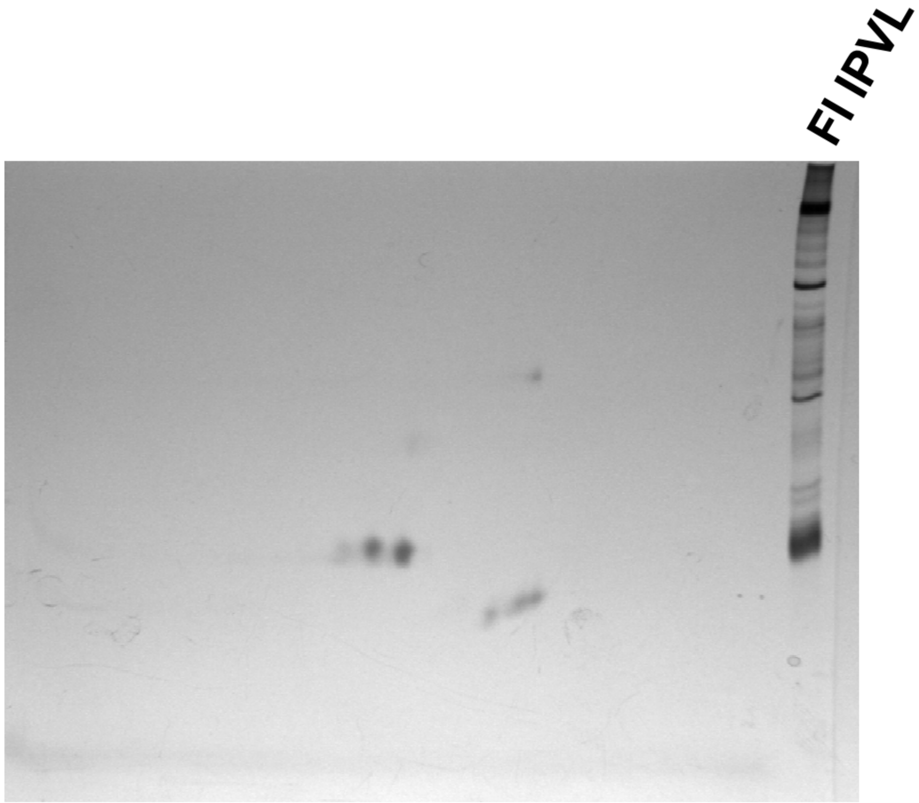

- An aliquote (10 μl) of dialyzed fraction 8 of the re-fractionation followed by the LP-IEF using carrier ampholyte for pH range of ~5–7 was subjected with the IEF markers to 2D-PAGE under non-reducing condition.
- Proteins in the 2D-gel were visualized by silver staining.
- Image of dried gel was obtained using LuminoGraph II (ATTO, Japan).

Original gel image for panel f of Fig6F and panel 9(f) of S5H Fig

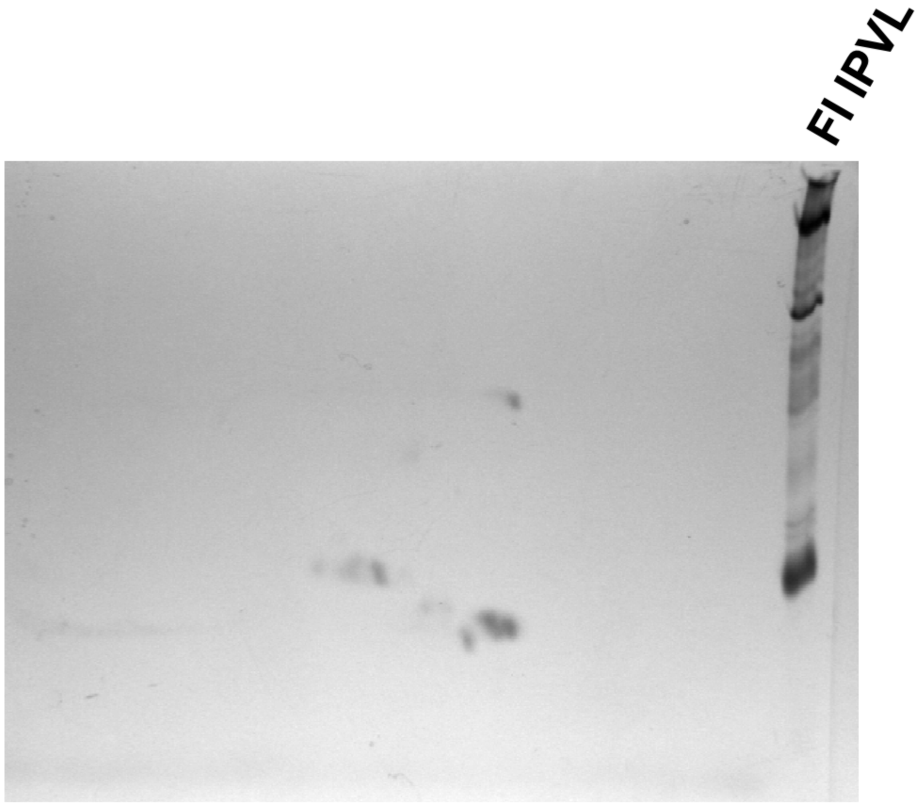

- An aliquote (10 μl) of dialyzed fraction 9 of the re-fractionation followed by the LP-IEF using carrier ampholyte for pH range of ~5–7 was subjected with the IEF markers to 2D-PAGE under non-reducing condition.
- Proteins in the 2D-gel were visualized by silver staining.
- Image of dried gel was obtained using LuminoGraph II (ATTO, Japan).

Original gel image for S5I Fig

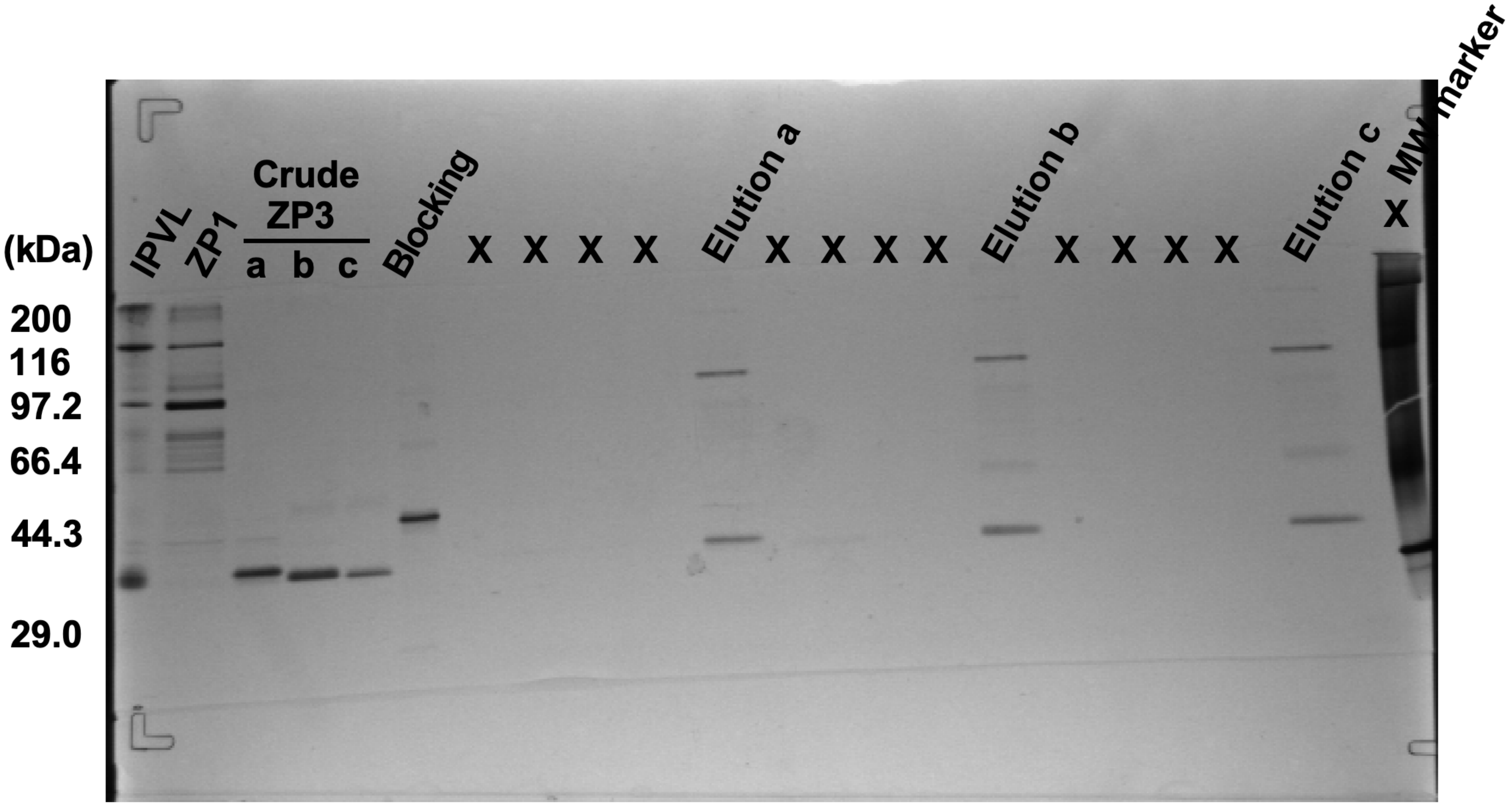

• The MW labeling depended on the comparison with S5D Fig.

Original blot image for
Fig6G and S6J Fig (anti-ZP3)

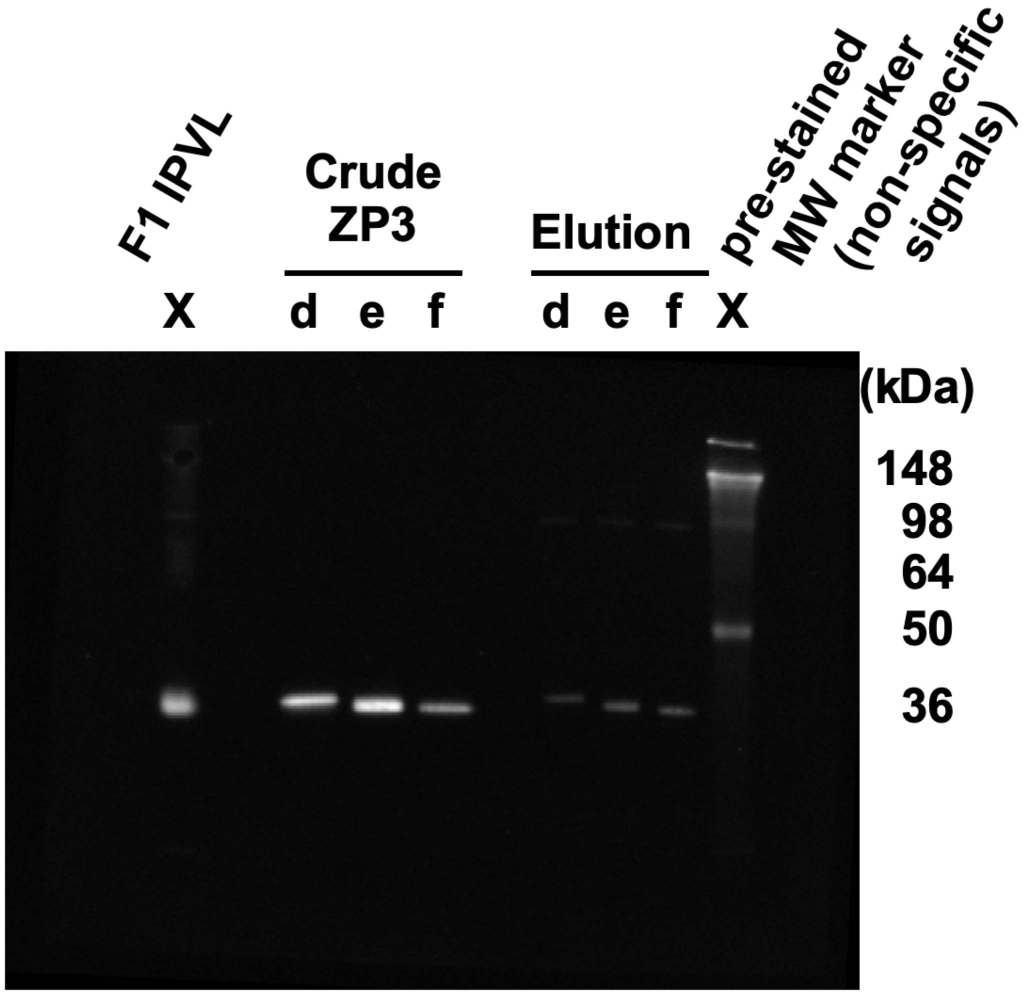

- The Crude ZP3-isoform fractions d–f and the corresponding elution fractions d–f were subjected to SDS-PAGE under non-reducing conditions.
- ZP3 in the gel were detected by Western blotting using the anti-ZP3 as a primary antibody.
- Image of blot was obtained using LuminoGraph II (ATTO, Japan).

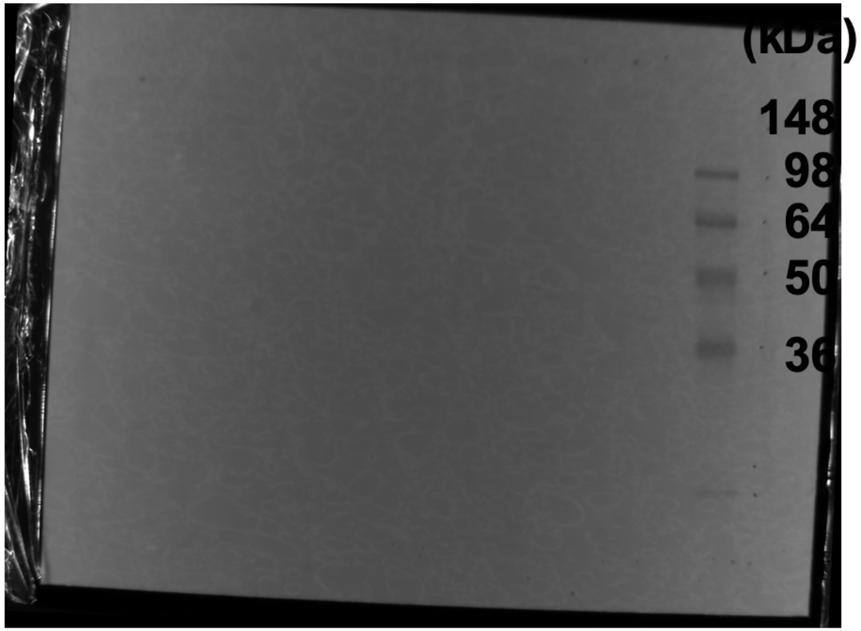

- Light -field image of the membrane.
  Band-positions of the pre-stained MW marker were labeled.
  Some of the markers were non-specifically detrected by Western blotting using anti-ZP3.

Original blot image for
Fig6G and S6J Fig (re-probed by anti-ZP1)

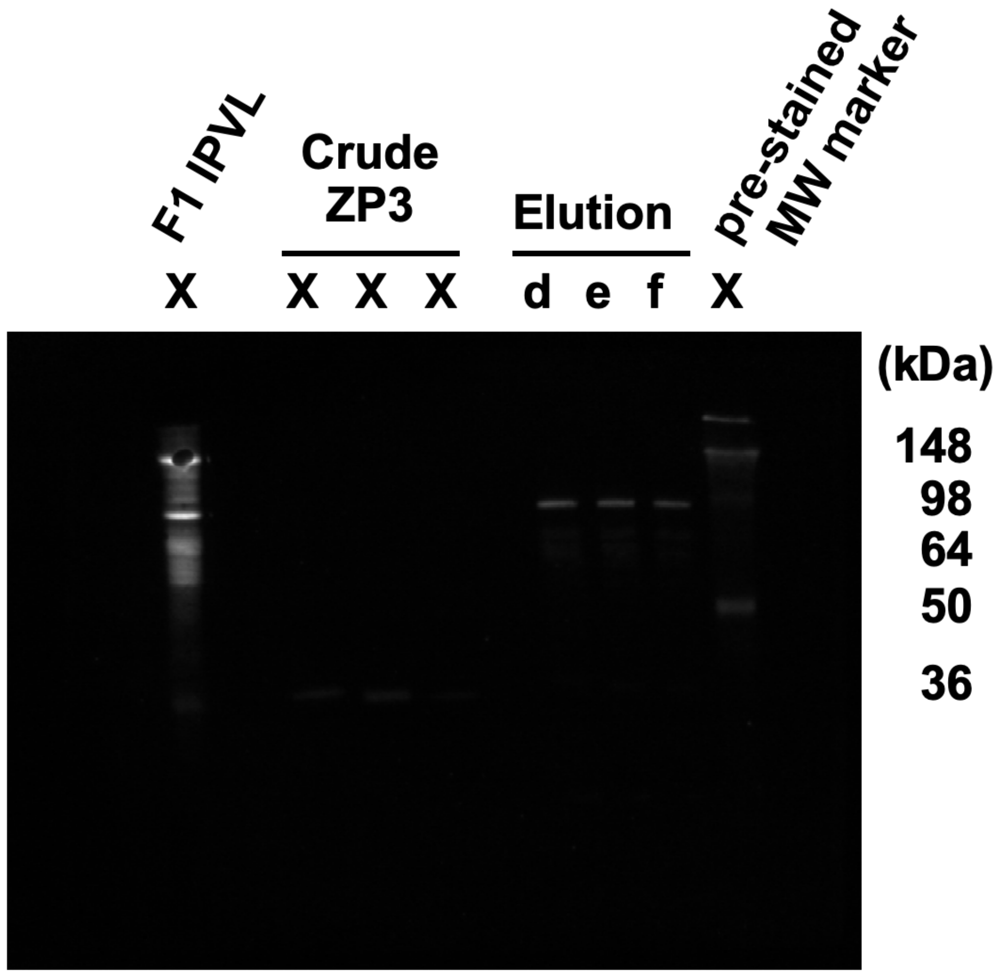

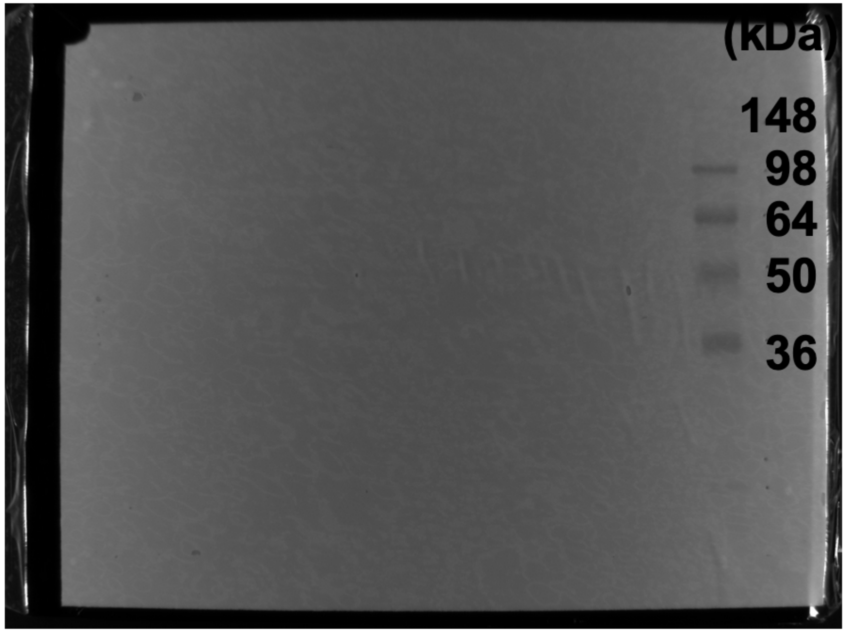

- The Crude ZP3-isoform fractions d–f and the corresponding elution fractions d–f were subjected to SDS-PAGE under non-reducing conditions.
- ZP1 in the gel were detected by re-probing of the membrane using the anti-ZP1 repeat as a primary antibody.
- Image of blot was obtained using LuminoGraph II (ATTO, Japan).

- Light -field image of the membrane.
  Band-positions of the pre-stained MW marker were labeled.
  Some of the markers were non-specifically detrected by Western blotting.
