# Supplementary material for: New insights into the role of microheterogeneity of ZP3 during structural maturation of the avian equivalent of mammalian zona pellucida
Source: PLoS One. 2023 Mar 21;18(3):e0283087. doi: 10.1371/journal.pone.0283087 (PMC10030024; doi:10.1371/journal.pone.0283087)
Supplement: Attachment — Submitted filename: 2022 Rebuttal letter for PLOS ONE Okumura.docx [file pone.0283087.s008.docx]

Thank you for reviewing our manuscript entitled “New Insights into the Role of Microheterogeneity of ZP3 During Structural Maturation of the Avian Equivalent of Mammalian Zona Pellucida”. According to reviewers’ comments, we revised our manuscript, and made answers as follows.

Answers to the questions:

Introduction section:

>It is better to include enough background information, rationale, hypothesis, and objectives of this study for better readability. Line 100-111 explains the results. Authors may consider modifying these statements.

>Why is this study important, and how will it help the scientific community in the field of poultry science or biology?

We added sentences “Therefore, understanding the mechanisms of egg-coat formation could provide the basis for developing new approaches to control human and animal fertility including new therapies for infertility that might be related to the egg-coat abnormality.” in lines 63–65, “…. we aimed to investigate the process of egg-coat matrix formation from ZP glycoproteins using the chicken model. For this purpose, we initially collected chicken IPVLs….” in lines 102–103, and “Our results will be useful for understanding the egg-coat formation mechanisms.” in lines 118–119 of both the Revised Manuscript with Track Changes and the Manuscript.

We left line 100–111 of the initial manuscript in the revised one because this part may be useful to understand the complicated results of this study.

Materials and Methods:

>Line 118-119: Although ovary and blood were collected from a local chicken meat processor, the sampling procedure is unclear and needs a clear explanation. The number of hens, sampling, and handling procedures are missing. As this is egg-type chicken, why were samples collected from meat processors?

We replaced the word “meat processor” to more suitable one “slaughter plant” in line 127, and added sentences “In this plant, large number of White-Leghorn hens (~15,000 hens/day) are killed by bleeding from carotid artery using a mechanical knife in an automated line system and slaughtered by hand followed by a brief scalding in ~63°C of hot water for 80 seconds to be remove feathers. The blood and ovary were collected immediately and transported to our laboratory in an ice box.” in lines 127–131 of the Revised Manuscript with Track Changes and lines 127–130 of the Manuscript.

>Line 131-133: Approximately 30 ovarian follicles for each growth stage of the yellow follicles were collected from 3 kg of 132 the ovaries of the commercial White-Leghorn hens. This statement is confusing.

We replaced the sentence “Approximately 30 ovarian follicles for each growth stage of the yellow follicles were collected from 3 kg of 132 the ovaries of the commercial White-Leghorn hens,” to the one “Respective approximately 30 of yellow follicles at each growth stage (F1–F3) were collected from 3 kg of the ovaries of the commercial White-Leghorn hens,” in lines 143–144 of both the Revised Manuscript with Track Changes and the Manuscript.

>Figures 1 A, B, and C look blurry, and difficult to understand. Authors may indicate with arrows or other simpler ways to understand for the readers. Same comments for Figure 2.

We revised Figures 1 and 2 by mainly drawing elliptic arrows indicating rotation angles of the images. We also corrected or added some annotation words in the Results section and Figure legends, correspondingly.

>Figure legends (Fig 1-7) are missing. The authors have only provided the Supplementary figure legends. It’s very hard to evaluate the results without figure legends.

According to the instructions for authors to prepare manuscript, the figure captions and legends should appear directly after the paragraph in which they are first cited. We followed the instruction and did not put the Figure legends section in the initial manuscript. However, the location of Figure legend of Fig2 was not suitable, and there were 2 copies of Figure legend of Fig6 in the initial manuscript, these mistakes are corrected in the Revised Manuscript with Track Changes and the Manuscript.

Other revised points:

The mouse antisera were raised against recombinant domains of chicken ZP1 that were derived from our originally cloned cDNA of chicken ZP1. Therefore, we mentioned accession number of the cDNA in lines 170–172 of the Revised Manuscript with Track Changes and lines 169–171 of the Manuscript.

We had not mention how to assign the ZP3 spots that were separated by LP-IEF and detected in the 2D-PAGE gels to each ZP3 isoforms in the initial manuscript. Therefore, we added the sentence “ZP3 spots detected in the 2D-PAGE gels were assigned to each ZP3 isoforms based on the aforementioned IEF markers and/or comparison of the spot patterns to that of ZP3 isoforms in the F1 IPVL.” in lines 256–257 of the Revised Manuscript with Track Changes and lines 255–256 of the Manuscript.

Current affiliation of the author, Shunsuke Nishio in line 9, was added in lines 25–26.
