# Supplementary material for: New insights into the role of microheterogeneity of ZP3 during structural maturation of the avian equivalent of mammalian zona pellucida
Source: PLoS One. 2023 Mar 21;18(3):e0283087. doi: 10.1371/journal.pone.0283087 (PMC10030024; doi:10.1371/journal.pone.0283087)
Supplement: Attachment — Submitted filename: 2022 Rebuttal letter for PLOS ONE Okumura rev-2.docx [file pone.0283087.s009.docx]

Thank you for reviewing our manuscript entitled “New Insights into the Role of Microheterogeneity of ZP3 During Structural Maturation of the Avian Equivalent of Mammalian Zona Pellucida”. According to reviewers’ comments, we revised our manuscript, and made answers as follows.

Answers to the question:

> It is also not clear about the sampling. The authors mentioned that the blood and ovary were collected immediately. Does it mean that samples were collected immediately before scalding in ~63°C of hot water for 80 seconds? Please make these statements clear.

The sentence “The blood and ovary were collected immediately and transported to our laboratory in an ice box.” in lines 130–131 of the Revised Manuscript with Track Changes was replaced with “The blood and ovary were collected during the bleeding and immediately after the removal of feathers, respectively, and transported to our laboratory in an ice box.” in lines 129–131 of the Manuscript. Ovaries of chicken are deeply encased in the body cavity being surrounded by thick adipose tissues, and therefore, denaturation of the egg coat must not be caused by the brief scalding in ~63°C of hot water for 80 seconds.

Other revised points 1) to 17) including corrections of English grammatical errors:

1) The phrase “with approval from the committee” in line 126 of the Revised Manuscript with Track Changes was deleted in line 126 of the Manuscript to correct a grammatical error.

2) The phrase “with approval from the committee” in line 135 of the Revised Manuscript with Track Changes was replaced with “with the approval” in line 135 of the Manuscript to avoid repetition.

3) The phrase “in Figs 1 and Supplement Figs S1” in line 308 of the Revised Manuscript with Track Changes was replaced with “in Fig 1 and S1 Fig” in line 307 of the Manuscript to meet PLOS ONE’s style.

4) The sentence “Proportions of the signal intensities from ZP3 isoforms 3 to 7 are compared between F1 IPVLs of the commercial White-Leghorn and WL-G hens, F1 and F2, F2 and F3, and F1 and F3 IPVLs of the commercial White-Leghorn hens are compared by chi-square test (P-values on the right side of the graphs).” in lines 430–433 of the Revised Manuscript with Track Changes was replaced with “Proportions of the signal intensities from ZP3 isoforms 3 to 7 are compared between F1 IPVLs of the commercial White-Leghorn and WL-G hens, and between F1 and F2, F2 and F3, and F1 and F3 IPVLs of the commercial White-Leghorn hens, respectively, by chi-square test (P-values on the right side of the graphs).” in lines 428–431 of the Manuscript to improve the readability and to correct a grammatical error.

5) The word “were” in line 518 of the Revised Manuscript with Track Changes was replaced with “are” in line 516 of the Manuscript to correct the tense.

6) The phrase “*lanes* d to f” in line 534 of the Revised Manuscript with Track Changes was replaced with “*lanes* d–f” in line 532 of the Manuscript to use the same notation.

7) The phrase “already there are distinct differences” in lines 616–617 of the Revised Manuscript with Track Changes was replaced with “there already be distinct differences” in lines 614–615 of the Manuscript to correct a grammatical error.

8) The phrase “and F1 follicles (Fig 2B and S2D Fig)” in line 654 of the Revised Manuscript with Track Changes was replaced with “and F1 (Fig 2B and S2D Fig) follicles” in line 652 of the Manuscript to improve the readability.

9) The phrase “These results suggest that” in lines 659–660 of the Revised Manuscript with Track Changes was replaced with “These observations suggest that” in lines 657–658 of the Manuscript.

10) The phrase “the ZP3 isoforms 3–7” in lines 673–674 of the Revised Manuscript with Track Changes was replaced with “the ZP3 isoforms 3 to 7” in lines 670–671 of the Manuscript to use the same notation.

11) The phrase “these data suggested that the expression levels of individuals ZP3 isoforms” in lines 684–685 of the Revised Manuscript with Track Changes was replaced with “these data suggest that the expression levels of individual ZP3 isoforms” in lines 681–682 of the Manuscript to correct the tense and a grammatical error.

12) The phrase “that are associated with” in line 787 of the Revised Manuscript with Track Changes was replaced with “that might be associated with” in line 784 of the Manuscript.

13) The phrase “in the similar format as Fig 1” in line 925 of the Revised Manuscript with Track Changes was replaced with “in the same format as Fig 1” in line 922 of the Manuscript.

14) The phrase “in both *B* and *C*” in line 926 of the Revised Manuscript with Track Changes was replaced with “in both B and C” in line 923 of the Manuscript to correct styles of A and B.

15) The phrase “indicated by *white elliptic arrows*” in line 926 of the Revised Manuscript with Track Changes was replaced with “(indicated by *white elliptic arrows*)” in line 923 of the Manuscript to improve the readability using parentheses.

16) The phrase “in the similar format as Fig 2” in line 934 of the Revised Manuscript with Track Changes was replaced with “in the same format as Fig 2” in line 931 of the Manuscript.

17) The phrase “*white dotted lines*” in line 934 of the Revised Manuscript with Track Changes was replaced with “*gray dotted lines*” in line 931 of the Manuscript.
