# Supplementary material for: New insights into the role of microheterogeneity of ZP3 during structural maturation of the avian equivalent of mammalian zona pellucida
Source: PLoS One. 2023 Mar 21;18(3):e0283087. doi: 10.1371/journal.pone.0283087 (PMC10030024; doi:10.1371/journal.pone.0283087)
Supplement: Attachment — Submitted filename: 2022 Rebuttal letter for PLOS ONE Okumura rev-3.docx [file pone.0283087.s011.docx]

Thank you for the detailed reviewing our manuscript entitled “New Insights into the Role of Microheterogeneity of ZP3 During Structural Maturation of the Avian Equivalent of Mammalian Zona Pellucida”. According to academic editor’s comments, we confirmed and corrected 144 points of writing errors including your corrections as follows.

Corrections of writing errors:

> The authors have responded to all the comments, however, there are several writing errors that I had pointed out in the previous version. I have edited/corrected most of the errors and suggested to chem if any errors (see ATTACHED file). Authors need to check further and confirm if there are any writing errors. After confirming the writing error, the manuscript will be acceptable for publication.

1) A definite article was inserted into “during structural maturation” in line 41 of the Revised Manuscript with Track Changes to be “during the structural maturation” in line 41 of the Manuscript.

2) A comma was inserted into “ZP1, ZP2, ZP3, ZP4, ZPD and ZPAX” in line 52 of the Revised Manuscript with Track Changes to be “ZP1, ZP2, ZP3, ZP4, ZPD, and ZPAX” in line 52 of the Manuscript.

3) A dash in “the sperm–egg” in line 53 of the Revised Manuscript with Track Changes was replaced with a hyphen to be “the sperm-egg” in line 53 of the Manuscript.

4) A comma was inserted into “interactions including” in line 54 of the Revised Manuscript with Track Changes to be “interactions, including” in line 54 of the Manuscript.

5) A comma was inserted into “*mZP1*^–/–^, *mZP2*^–/–^ and *mZP3*^–/–^” in line 57 of the Revised Manuscript with Track Changes to be “*mZP1*^–/–^, *mZP2*^–/–^, and *mZP3*^–/–^” in line 57 of the Manuscript.

6) A definite article and a comma were inserted into “ZP1, ZP2 or ZP3 gene” and the singular form was replaced with a plural form in “ZP1, ZP2 or ZP3 gene” in line 59 of the Revised Manuscript with Track Changes to be “the ZP1, ZP2, or ZP3 genes” in line 59 of the Manuscript.

7) A dash in “the sperm–egg” in line 61 of the Revised Manuscript with Track Changes was replaced with a hyphen to be “the sperm-egg” in line 61 of the Manuscript.

8) A comma was inserted into “fertility including” in line 64 of the Revised Manuscript with Track Changes to be “fertility, including” in line 64 of the Manuscript.

9) The phrase “For elucidating these mechanisms” in lines 67 to 68 of the Revised Manuscript with Track Changes was replaced with “To elucidate these mechanisms” in lines 67 to 68 of the Manuscript.

10) A definite article and a comma were inserted into “characterize structural, biochemical and physiological” in lines 68 to 69 of the Revised Manuscript with Track Changes to be “characterize the structural, biochemical, and physiological” in line 68 of the Manuscript.

11) The phrase “samples that are collected from” in line 70 of the Revised Manuscript with Track Changes was replaced with “samples collected from” in lines 69 to 70 of the Manuscript.

12) A comma was inserted into “poultry species including” in line 75 of the Revised Manuscript with Track Changes to be “poultry species, including” in line 74 of the Manuscript.

13) A comma in “formed quickly,” in line 76 of the Revised Manuscript with Track Changes was deleted to be “formed quickly” in line 76 of the Manuscript.

14) A comma was inserted into “F3, F2 and F1” in line 104 of the Revised Manuscript with Track Changes to be “F3, F2, and F1” in line 104 of the Manuscript.

15) An indefinite article was inserted into “in few days” in line 108 of the Revised Manuscript with Track Changes to be “in a few days” in line 108 of the Manuscript.

16) A definite article was inserted into “process of egg-coat matrix” in line 117 of the Revised Manuscript with Track Changes to be “process of the egg-coat matrix” in line 117 of the Manuscript.

17) A definite article in “during the ovarian follicle” in line 118 of the Revised Manuscript with Track Changes was deleted to be “during ovarian follicle” in line 118 of the Manuscript.

18) A comma was inserted into “slaughtered by hand” in line 128 of the Revised Manuscript with Track Changes to be “slaughtered by hand,” in line 128 of the Manuscript.

19) The phrase “to be remove feathers” in line 129 of the Revised Manuscript with Track Changes was replaced with “to remove feathers” in line 129 of the Manuscript.

20) A definite article was inserted into “guidelines of Meijo University” in line 134 of the Revised Manuscript with Track Changes to be “guidelines of the Meijo University” in line 134 of the Manuscript.

21) A definite article in “with the approval” in line 135 of the Revised Manuscript with Track Changes was deleted to be “with approval” in line 135 of the Manuscript.

22) A comma was inserted into “Respective approximately” in line 143 of the Revised Manuscript with Track Changes to be “Respective, approximately” in line 143 of the Manuscript.

23) A definite article was inserted into “cloned into pGEM-T easy vector” in line 157 of the Revised Manuscript with Track Changes to be “cloned into the pGEM-T easy vector” in line 157 of the Manuscript.

24) The phrase “IPVLs that were isolated from” in line 164 of the Revised Manuscript with Track Changes was replaced with “IPVLs isolated from” in line 164 of the Manuscript.

25) A definite article in “in the non-reducing conditions” in line 192 of the Revised Manuscript with Track Changes was deleted to be “in non-reducing conditions” in line 192 of the Manuscript.

26) A comma was inserted into “(CBB) staining and” in line 194 of the Revised Manuscript with Track Changes to be “(CBB) staining, and” in line 194 of the Manuscript.

27) A comma was inserted into “±0.4, ±0.5, ±1.0 or ±1.1 Da” in line 231 of the Revised Manuscript with Track Changes to be “±0.4, ±0.5, ±1.0, or ±1.1 Da” in line 231 of the Manuscript.

28) The phrase “the rest of fractions” in line 248 of the Revised Manuscript with Track Changes was replaced with “the remaining fractions” in line 248 of the Manuscript.

29) A definite article was inserted into “Examination of binding capacity” in line 258 of the Revised Manuscript with Track Changes to be “Examination of the binding capacity” in line 258 of the Manuscript.

30) A definite article was inserted into “pulled by magnetic field” in line 264 of the Revised Manuscript with Track Changes to be “pulled by the magnetic field” in line 264 of the Manuscript.

31) An indefinite article was inserted into “pulled by magnetic field” in line 266 of the Revised Manuscript with Track Changes to be “pulled by a magnetic field” in line 266 of the Manuscript.

32) A comma was inserted into “(Merck, Darmstadt, Germany) being transformed” in line 278 of the Revised Manuscript with Track Changes to be “(Merck, Darmstadt, Germany), being transformed” in line 278 of the Manuscript.

33) An indefinite article was inserted into “pulled by magnetic field” in line 289 of the Revised Manuscript with Track Changes to be “pulled by a magnetic field” in line 289 of the Manuscript.

34) An indefinite article was inserted into “pulled by magnetic field” in line 291 of the Revised Manuscript with Track Changes to be “pulled by a magnetic field” in line 291 of the Manuscript.

35) An indefinite article was inserted into “pulled by magnetic field” in lines 293 to 294 of the Revised Manuscript with Track Changes to be “pulled by a magnetic field” in lines 293 to 294 of the Manuscript.

36) The phrase “the 3D Viewer plug in” in line 299 of the Revised Manuscript with Track Changes was replaced with “the 3D Viewer plugin” in line 299 of the Manuscript.

37) A comma in “the Sigma settings of 0.001),” in line 303 of the Revised Manuscript with Track Changes was deleted to be “the Sigma settings of 0.001)” in line 303 of the Manuscript.

38) A definite article in “the all analyzed signals” in line 310 of the Revised Manuscript with Track Changes was deleted to be “all analyzed signals” in line 310 of the Manuscript.

39) A definite article in “the Cochran's rule” in line 313 of the Revised Manuscript with Track Changes was deleted to be “Cochran's rule” in lines 312 to 313 of the Manuscript.

40) A comma was inserted into “S2A–S2C Figs) and” in line 324 of the Revised Manuscript with Track Changes to be “S2A–S2C Figs), and” in line 324 of the Manuscript.

41) A comma was inserted into “panels 2, 2–3 or 2–4” in line 329 of the Revised Manuscript with Track Changes to be “panels 2, 2–3, or 2–4” in line 329 of the Manuscript.

42) A comma was inserted into “F2 (B) and F1 (C)” in line 339 of the Revised Manuscript with Track Changes to be “F2 (B), and F1 (C)” in line 329 of the Manuscript.

43) A comma was inserted into “150° and 180°” in line 343 of the Revised Manuscript with Track Changes to be “150°, and 180°” in line 343 of the Manuscript.

44) The phrase “cross section” in line 347 of the Revised Manuscript with Track Changes was replaced with “the cross-section” in line 347 of the Manuscript.

45) A comma was inserted into “the Fig 1 except that” in line 353 of the Revised Manuscript with Track Changes to be “the Fig 1, except that” in line 353 of the Manuscript.

46) The phrase “inside the *panels* 1” in line 357 of the Revised Manuscript with Track Changes was replaced with “inside *panel* 1” in line 357 of the Manuscript.

47) A comma in “(*panels* 2 and 3 in A, and *panel* 2 in B)” in line 361 of the Revised Manuscript with Track Changes was deleted to be “(*panels* 2 and 3 in A and *panel* 2 in B)” in line 361 of the Manuscript.

48) The plural form was replaced with a singular form in “one surfaces of the IPVLs” in line 364 of the Revised Manuscript with Track Changes to be “one surface of the IPVLs” in line 59 of the Manuscript.

49) A comma was inserted into “the F3, F2 and F1 follicles” in line 368 of the Revised Manuscript with Track Changes to be “the F3, F2, and F1 follicles” in line 368 of the Manuscript.

50) A comma was inserted into “~2, ~7 and ~7 µm” in line 368 of the Revised Manuscript with Track Changes to be “~2, ~7, and ~7 µm” in line 368 of the Manuscript.

51) A comma was inserted into “the F3, F2 and F1 follicles” in line 369 of the Revised Manuscript with Track Changes to be “the F3, F2, and F1 follicles” in line 369 of the Manuscript.

52) A comma was inserted into “1.4 ± 0.30 and 1.5 ± 0.58 µm” in lines 369 to 370 of the Revised Manuscript with Track Changes to be “1.4 ± 0.30, and 1.5 ± 0.58 µm” in lines 369 to 370 of the Manuscript.

53) The phrase “similar to each other, a number of holes” in line 371 of the Revised Manuscript with Track Changes was replaced with “similar, several holes” in line 371 of the Manuscript.

54) A comma was inserted into “*panels* 10 and 11 in Fig 1B, S1B and S1C Figs” in line 373 of the Revised Manuscript with Track Changes to be “*panels* 10 and 11 in Fig 1B, S1B, and S1C Figs” in line 373 of the Manuscript.

56) A definite article in “cross sections of the *panels* 9” in line 373 of the Revised Manuscript with Track Changes was deleted to be “cross sections of *panels* 9” in line 373 of the Manuscript.

57) A comma was inserted into “15°, 0° and 30°” in line 374 of the Revised Manuscript with Track Changes to be “15°, 0°, and 30°” in line 374 of the Manuscript.

58) The phrase “between the surface layers” in line 380 of the Revised Manuscript with Track Changes was replaced with “between their surface layers” in line 380 of the Manuscript.

59) A definite article in “in the *panel* 1” in line 381 of the Revised Manuscript with Track Changes was deleted to be “in *panel* 1” in line 381 of the Manuscript.

60) A comma was inserted into “protrusions including” in line 385 of the Revised Manuscript with Track Changes to be “protrusions, including” in line 385 of the Manuscript.

61) A definite article in “including the both surface” in line 385 of the Revised Manuscript with Track Changes was deleted to be “including both surface” in line 385 of the Manuscript.

62) A definite article was inserted into “deeper region” in line 389 of the Revised Manuscript with Track Changes to be “the deeper region” in line 388 of the Manuscript.

63) The phrase “IPVLs that were collected from” in line 412 of the Revised Manuscript with Track Changes was replaced with “IPVLs collected from” in line 412 of the Manuscript.

64) The phrase “proteins that were also detected” in line 416 of the Revised Manuscript with Track Changes was replaced with “proteins also detected” in line 416 of the Manuscript.

65) The phrase “similarly to the *panel* 1, although ranging from” in line 419 of the Revised Manuscript with Track Changes was replaced with “similarly to *panel* 1, ranging from” in line 419 of the Manuscript.

66) A definite article in “similarly to the *panel* 3” in line 421 of the Revised Manuscript with Track Changes was deleted to be “similarly to *panel* 3” in line 421 of the Manuscript.

67) The phrase “shown on the bottom of the *graph* d” in line 427 of the Revised Manuscript with Track Changes was replaced with “shown at the bottom of *graph* d” in lines 426 to 427 of the Manuscript.

68) The phrase “although that were” in line 442 of the Revised Manuscript with Track Changes was replaced with “although they were” in line 441 of the Manuscript.

69) The word “obtained” in line 451 of the Revised Manuscript with Track Changes was deleted in line 450 of the Manuscript.

70) A definite article was inserted into “in S1 Table” in line 462 of the Revised Manuscript with Track Changes to be “in the S1 Table” in line 461 of the Manuscript.

71) The phrase “the peptides that are detected” in line 473 of the Revised Manuscript with Track Changes was replaced with “the peptides detected” in line 472 of the Manuscript.

72) The phrase “residues that contain the identified modifications” in line 474 of the Revised Manuscript with Track Changes was replaced with “residues containing the identified modifications” in line 473 of the Manuscript.

73) The phrase “mutants being derived from” in line 475 of the Revised Manuscript with Track Changes was replaced with “mutants derived from” in line 474 of the Manuscript.

74) A comma was inserted into “318/320–329 and 330–347” in line 486 of the Revised Manuscript with Track Changes to be “318/320–329, and 330–347” in line 485 of the Manuscript.

75) A comma was inserted into “318/320–329 and 330–347” in line 487 of the Revised Manuscript with Track Changes to be “318/320–329, and 330–347” in line 486 of the Manuscript.

76) A comma was inserted into “D33, A36 and A41” in line 494 of the Revised Manuscript with Track Changes to be “D33, A36, and A41” in line 493 of the Manuscript.

77) A comma was inserted into “N339 and the combination of” in line 497 of the Revised Manuscript with Track Changes to be “N339, and the combination of” in line 496 of the Manuscript.

78) The phrase “histidine residues, which majority of them are” in line 511 of the Revised Manuscript with Track Changes was replaced with “histidine residues, most of which are” in line 510 of the Manuscript.

79) The phrase “26 to 29 of histidine residues” in lines 512 to 513 of the Revised Manuscript with Track Changes was replaced with “26 to 29 histidine residues” in line 511 of the Manuscript.

80) A definite article was inserted into “Migration position” in line 533 of the Revised Manuscript with Track Changes to be “The migration position” in line 532 of the Manuscript.

81) The phrase “the fractions that were obtained from” in line 545 of the Revised Manuscript with Track Changes was replaced with “the fractions obtained from” in line 544 of the Manuscript.

82) A comma was inserted into “the fractions 3, 7 and 10” in line 547 of the Revised Manuscript with Track Changes to be “the fractions 3, 7, and 10” in line 546 of the Manuscript.

83) A comma was inserted into “5 to 7 and 7 to 8” in line 548 of the Revised Manuscript with Track Changes to be “5 to 7, and 7 to 8” in line 547 of the Manuscript.

84) The phrase “the fractions that were obtained from” in line 550 of the Revised Manuscript with Track Changes was replaced with “the fractions obtained from” in line 549 of the Manuscript.

85) A comma was inserted into “the fractions 6, 8 and 9” in lines 552 to 553 of the Revised Manuscript with Track Changes to be “the fractions 6, 8, and 9” in line 551 of the Manuscript.

86) A comma was inserted into “isoforms 5, 6 to 7 and 7” in line 553 of the Revised Manuscript with Track Changes to be “isoforms 5, 6 to 7, and 7” in line 552 of the Manuscript.

87) A comma was inserted into “fractions d, e and f” in line 554 of the Revised Manuscript with Track Changes to be “fractions d, e, and f” in line 553 of the Manuscript.

88) A typo “re-fractionaton” in line 566 of the Revised Manuscript with Track Changes was replaced with “re-fractionation in line 564 of the Manuscript.

89) A comma was inserted into “the fractions 3, 7 and 10” in line 567 of the Revised Manuscript with Track Changes to be “the fractions 3, 7, and 10” in line 565 of the Manuscript.

90) A comma was inserted into “fractions a, b and c” in line 568 of the Revised Manuscript with Track Changes to be “fractions a, b, and c” in line 565 of the Manuscript.

91) A comma was inserted into “the fractions 6, 8 and 9” in line 568 of the Revised Manuscript with Track Changes to be “the fractions 6, 8, and 9” in line 566 of the Manuscript.

92) A comma was inserted into “fractions d, e and f” in line 569 of the Revised Manuscript with Track Changes to be “fractions d, e, and f” in lines 566 to 567 of the Manuscript.

93) A definite article was inserted into “the top of *bars*” in line 578 of the Revised Manuscript with Track Changes to be “the top of the *bars*” in line 576 of the Manuscript.

94) A comma was inserted into “*single*, *double* and *triple asterisks*” in line 587 of the Revised Manuscript with Track Changes to be “*single*, *double*, and *triple asterisks*” in line 585 of the Manuscript.

95) A comma in “Whereas, that” in line 596 of the Revised Manuscript with Track Changes was deleted to be “Whereas that” in line 594 of the Manuscript.

96) The phrase “Taken together, these results” in lines 598 to 599 of the Revised Manuscript with Track Changes was replaced with “These results” in lines 596 to 597 of the Manuscript.

97) The phrase “isoform 7 by itself exhibited” in line 599 of the Revised Manuscript with Track Changes was replaced with “isoform 7 exhibited” in line 597 of the Manuscript.

98) A definite article in “there might be the possible synergistic effects” in line 600 of the Revised Manuscript with Track Changes was deleted to be “there might be possible synergistic effects” in line 598 of the Manuscript.

99) A comma was inserted into “egg coat including” in line 607 of the Revised Manuscript with Track Changes to be “egg coat, including” in line 604 of the Manuscript.

100) Commas were inserted into “(IPVL) and so on is” in line 608 of the Revised Manuscript with Track Changes to be “(IPVL), and so on, is” in line 605 of the Manuscript.

101) The phrase “showed that there are distinct differences” in line 609 of the Revised Manuscript with Track Changes was replaced with “showed distinct differences” in line 606 of the Manuscript.

102) An indefinite article was inserted into “is composed of more robust” in line 613 of the Revised Manuscript with Track Changes to be “is composed of a more robust” in line 610 of the Manuscript.

103) The phrase “there already be distinct” in line 616 of the Revised Manuscript with Track Changes was replaced with “there are distinct” in line 613 of the Manuscript.

104) An indefinite article was inserted into “one surface has smooth” in line 617 of the Revised Manuscript with Track Changes to be “one surface has a smooth” in line 614 of the Manuscript.

105) A comma was inserted into “*panels* 1, 2, 5 and 6” in line 618 of the Revised Manuscript with Track Changes to be “*panels* 1, 2, 5, and 6” in line 615 of the Manuscript.

106) A comma was inserted into “*panels* 3, 4, 7 and 8” in line 619 of the Revised Manuscript with Track Changes to be “*panels* 3, 4, 7, and 8” in line 616 of the Manuscript.

107) A definite article was inserted into “formation of egg-coat matrix” in line 620 of the Revised Manuscript with Track Changes to be “formation of the egg-coat matrix” in line 617 of the Manuscript.

108) A comma was inserted into “ZP1, ZP3 and ZPD” in line 625 of the Revised Manuscript with Track Changes to be “ZP1, ZP3, and ZPD” in lines 621 to 622 of the Manuscript.

109) The phrase “interestingly, there are some cavities and tunnels traversing” in lines 633 to 634 of the Revised Manuscript with Track Changes was replaced with “interestingly, some cavities and tunnels are traversing” in lines 630 to 631 of the Manuscript.

110) A comma was inserted into “Fig 1B, S1B, S1C and S1E Figs” in line 644 of the Revised Manuscript with Track Changes to be “Fig 1B, S1B, S1C, and S1E Figs” in line 641 of the Manuscript.

111) A comma was inserted into “the F2 IPVLs suggest that” in line 648 of the Revised Manuscript with Track Changes to be “the F2 IPVLs, suggest that” in line 645 of the Manuscript.

112) A comma was inserted into “*panels* 3, 4, 7 and 8” in line 659 of the Revised Manuscript with Track Changes to be “panels 3, 4, 7, and 8” in line 656 of the Manuscript.

113) Commas were inserted into “proteins including chicken ZP3 are” in line 669 of the Revised Manuscript with Track Changes to be “proteins, including chicken ZP3, are” in line 666 of the Manuscript.

114) The phrase “Of note, the relative proportions” in line 672 of the Revised Manuscript with Track Changes was replaced with “Notably, the relative proportions” in line 669 of the Manuscript.

115) The phrase “In our previous study, it is suggested that” in line 689 of the Revised Manuscript with Track Changes was replaced with “Our previous study suggests that” in line 669 of the Manuscript.

116) A comma was inserted into “some modifications including” in line 692 of the Revised Manuscript with Track Changes to be “some modifications, including” in line 689 of the Manuscript.

117) A comma was inserted into “partially conserved at least” in lines 706 to 707 of the Revised Manuscript with Track Changes to be “partially conserved, at least” in line 703 of the Manuscript.

118) The phrase “chicken IPVLs, while the chicken line-specific” in line 708 of the Revised Manuscript with Track Changes was replaced with “chicken IPVLs. In contrast, the chicken line-specific” in lines 704 to 705 of the Manuscript.

119) The phrase “each chicken line, although little is known” in line 709 of the Revised Manuscript with Track Changes was replaced with “each chicken line. However, little is known” in lines 705 to 706 of the Manuscript.

120) A definite article was inserted into “regulating physiological” in line 710 of the Revised Manuscript with Track Changes to be “regulating the physiological” in line 706 of the Manuscript.

121) A comma was inserted into “isoforms 3, 4 and 7” in line 711 of the Revised Manuscript with Track Changes to be “isoforms 3, 4, and 7” in lines 707 to 708 of the Manuscript.

122) A comma was inserted into “isoforms 3, 4 and 7” in line 715 of the Revised Manuscript with Track Changes to be “isoforms 3, 4, and 7” in line 711 of the Manuscript.

123) A definite article was inserted into “Key findings of” in lines 759 to760 of the Revised Manuscript with Track Changes to be “The key findings of” in lines 755 to 756 of the Manuscript.

124) The phrase “isoform 7 by itself exhibits” in line 776 of the Revised Manuscript with Track Changes was replaced with “isoform 7 exhibits” in line 772 of the Manuscript.

125) A comma was inserted into “isoforms 3 to 7 at least in” in line 777 of the Revised Manuscript with Track Changes to be “isoforms 3 to 7, at least in” in line 773 of the Manuscript.

126) A comma was inserted into “granulosa cells although” in line 784 of the Revised Manuscript with Track Changes to be “granulosa cells, although” in line 780 of the Manuscript.

127) A comma was inserted into “F3 (A), F2 (B, C and E) and F1 (G) IPVLs” in lines 925 to 926 of the Revised Manuscript with Track Changes to be “F3 (A), F2 (B, C and E), and F1 (G) IPVLs” in lines 921 to 922 of the Manuscript.

128) The singular form was replaced with a plural form in “structure on IPVLs” in line 934 of the Revised Manuscript with Track Changes to be “structures on IPVLs” in line 930 of the Manuscript.

129) The phrase “in the same format as” in line 935 of the Revised Manuscript with Track Changes was replaced with “in the similar format as” in line 931 of the Manuscript.

130) The phrase “although all ranging from” in line 947 of the Revised Manuscript with Track Changes was replaced with “although all are ranging from” in line 943 of the Manuscript.

131) A definite article was inserted into “and positions of” in line 950 of the Revised Manuscript with Track Changes to be “and the positions of” in line 946 of the Manuscript.

132) A definite article in “immunoblot images of the Fig 5” in line 954 of the Revised Manuscript with Track Changes was deleted to be “immunoblot images of Fig 5” in line 950 of the Manuscript.

133) A comma was inserted into “fractions and” in line 955 of the Revised Manuscript with Track Changes to be “fractions, and” in line 951 of the Manuscript.

134) The phrase “fractions that were collected from” in line 958 of the Revised Manuscript with Track Changes was replaced with “fractions collected from” in line 954 of the Manuscript.

135) A definite article was inserted into “top of images” in line 960 of the Revised Manuscript with Track Changes to be “top of the images” in line 956 of the Manuscript.

136) The phrase “fractions 5 and 6 that are seen in A” in line 964 of the Revised Manuscript with Track Changes was replaced with “fractions 5 and 6 seen in A” in lines 959 to 960 of the Manuscript.

137) A definite article in “gel images of the Figs 6A” in line 965 of the Revised Manuscript with Track Changes was deleted to be “gel images of Figs 6A” in line 961 of the Manuscript.

138) A comma was inserted into “ZP3 and the MW markers” in line 966 of the Revised Manuscript with Track Changes to be “ZP3, and the MW markers” in line 962 of the Manuscript.

139) A comma was inserted into “ZP3 and the MW markers” in line 974 of the Revised Manuscript with Track Changes to be “ZP3, and the MW markers” in line 970 of the Manuscript.

140) A definite article in “blot images of the Figs 6C” in line 975 of the Revised Manuscript with Track Changes was deleted to be “blot images of Figs 6C” in line 971 of the Manuscript.

141) A comma was inserted into “ZP3 and the MW markers” in line 977 of the Revised Manuscript with Track Changes to be “ZP3, and the MW markers” in line 973 of the Manuscript.

142) A comma was inserted into “The *single*, *double* and *triple*” in line 977 of the Revised Manuscript with Track Changes to be “The *single*, *double*, and *triple*” in line 973 of the Manuscript.

143) The phrase “sequences being identified with” in line 981 of the Revised Manuscript with Track Changes was replaced with “sequences identified with” in line 977 of the Manuscript.

144) The phrase “peptides are shown by” in line 982 of the Revised Manuscript with Track Changes was replaced with “peptides are indicated by” in line 978 of the Manuscript.
