# Supplementary material for: New insights into the role of microheterogeneity of ZP3 during structural maturation of the avian equivalent of mammalian zona pellucida
Source: PLoS One. 2023 Mar 21;18(3):e0283087. doi: 10.1371/journal.pone.0283087 (PMC10030024; doi:10.1371/journal.pone.0283087)
Supplement: Attachment — Submitted filename: 2022 Rebuttal letter for PLOS ONE Okumura rev-4.docx [file pone.0283087.s012.docx]

Thank you for the detailed reviewing our manuscript entitled “New Insights into the Role of Microheterogeneity of ZP3 During Structural Maturation of the Avian Equivalent of Mammalian Zona Pellucida”. According to the academic editor’s and reviewers’ comments, we answered and corrected some points as follows.

**• For the comments from the reviewer #1**:

**Comment 1 from the reviewer #1**

> The reason using two different lines WL-G and commercial white leghorns was not clearly communicated. For that matter, the route of euthanasia for the WL-G lines was also not stated.

**(Answer)**

We used the WL-G (the long-term closed colony of White-Leghorn breed) hens in addition to the commercial White-Leghorn hens to demonstrate that the isoelectric-point microheterogeneity of chicken ZP3 is not due to the potential genetic heterogeneities in commercial hens. In general, chicken especially the commercial hens possess genetic heterogeneities, although we can prepare abundant IPVL from their large ovarian follicles. That is mentioned in lines 676 to 678 of both the Revised Manuscript with Track Changes and the Manuscript, although not clearly described.

To make the reason using the WL-G line and the route of euthanasia for the WL-G lines, we corrected the sentence “Fresh ovary and genomic DNA of the long-term closed colony of White-Leghorn breed (WL-G) hens were provided” was corrected into “To analyze the genetically homogeneous line of hen, fresh ovary and genomic DNA of the long-term closed colony of White-Leghorn breed (WL-G) hens that were killed similarly by hands were provided” in lines 131 to 133 of the Revised Manuscript with Track Changes and lines 131 to 132 of the Manuscript.

**Comment 2 from the reviewer #1**

> Lines 206-223: Was only one sample per ZP3 spot submitted for LC MADI MS/MS? If not, how many were submitted and was there any error among replicate sequences?

**(Answer)**

One sample per ZP3 spot was submitted for LC MALDI MS/MS. However, each spot of all the ZP3 isoforms 3 to 7 were obtained from both the F1 IPVL of commercial White-Leghorn and WL-G hens, and there are some similarities in the AA substitutions and modifications as mentioned in the manuscript.

**• For the comments from the reviewer #2:**

**Comment 1 from the reviewer #2**

> 1. The lack of reliable information on the orientation of the forming IPVL in the microscopy study is a major issue. The figures and results do not indicate whether the surfaces with 'smooth or homogeneously granular microstructures' (Fig. 1, panels 1, 2, 5, 6) and 'fibrous microstructures' (Fig. 1, panels 3, 4, 7, 8) are oriented towards the granulosa cells or towards the oocyte. Based on the presence of debris, the authors concluded that the "smooth surface of IPVL may be adjacent to the granulosa cell layer in the ovarian follicles" (line 637-638). I assume that the authors think that these debris come from the granulosa cells. But such debris could also be traces of yolk or remnants of the plasma membrane of the oocyte. The orientation needs to be defined more precisely and unambiguously. Is it possible to identify the orientation of the IPVL during the isolation step? This is very important to clarify because the predictive model for the structural maturation of the IPVL discussed in the manuscript strongly relies on the appearance of the surfaces and their orientation in the follicle. Moreover, it should be noted that some results in the literature tend to show the opposite. Indeed, in the fresh egg, the outer surface of the IPVL exposed after separation from the outer layer (i.e. the surface that was theoretically interacting with the granulosa cells) has thick fibers whereas the surface in contact with the yolk content or the oocyte has smaller fibers (In Kido and Doi, 1988, see Figure 4, panels C vs E, <https://doi.org/10.3382/ps.0670476>). In this case, the surface with the 'fibrous microstructures' could therefore be adjacent to the granulosa cells.

**(Answer)**

As the reviewer #2 pointed out, the presence of debris on the surface of IPVL with “smooth or homogeneously granular microstructures” will be not enough to indicate that this surface was adjacent to the granulosa cell layer in the ovarian follicles. Therefore, the phrase “, although further analyses to determine the orientation of IPVL on the oocyte are required” was added after the sentence “These results imply that the smooth surface of IPVL may be adjacent to the granulosa cell layer in the ovarian follicles.” in lines 641 to 642 of both the Revised Manuscript with Track Changes and the Manuscript.

In this study, we obtained the surface microstructure images of the IPVL from ovarian follicles under wet conditions as mentioned in the Materials and Method section. Therefore, our results are not directly comparable to the results of electron microscopic studies of IPVL. Furthermore, in the study the reviewer #2 cited, the specimen of IPVL were prepared from vitelline envelope of the fresh egg after the removal of the outer layer by SDS treatment, and the surface microstructure of the IPVL may be considerably denatured.

**Comment 2 from the reviewer #2**

> 2. The methodology used for the ZP1 binding assay raises many questions impairing the reliability of the results:

-The ZP1 binding test does not appear to be specific. The ZP3 crude extracts used are not pure and all bands present, including non-ZP3 bands, are found in the eluted fractions (see silver-stained gel, Fig. S5D).

**(Answer)**

Although the non-ZP3 proteins that may interact with the ZP1 beads are contained in the ZP3 crude extracts, the ZP1–ZP3 binding assay showed that there certainly be significant differences among the binding affinities of ZP3 isoforms against the ZP1 beads.

However, the phrase “, although the non-ZP3 contaminants in the crude ZP3-isoform fractions a–c (see Fig S5D) might interact with the ZP1-beads” was added after the sentence “The relative ratios of the ZP1 binding affinities of ZP3 in the fractions a–c (Fig 6D) showed that ZP3 in the mixture of ZP3 isoforms 5–7 (major components in the fraction b) bound to ZP1 with approximately 3-times higher affinity than ZP3 in the mixtures of ZP3 isoforms 2–4 and 7–8 (major components in the fractions a and c, respectively).” in lines 591 to 596 of both the Revised Manuscript with Track Changes and the Manuscript.

-The protein amounts/concentrations of ZP1 solution and ZP3 crude extracts used are not indicated in the material and method. To compare the binding capacity of different ZP3 fractions, ZP1 beads should normally be incubated with identical amounts of ZP3 protein, but immunoblot results suggest that the starting amounts are different for the different crude extracts used (Figs. 6C and 6G). To overcome this, the authors attempted to normalize their results with the ratio of the starting amount to the eluted amount of ZP3, but this method is not ideal. The presence of more or less abundant contaminating bands or proteins in the ZP3 fractions is problematic. Figures S5B and S5D show that ZP3 band is minor in fraction 10 (crude extract “c”). This fraction even seems to contain contaminating ZP1 protein, which can potentially bind ZP3 protein and prevent it from binding to the ZP1 beads.

**(Answer)**

In this ZP1–ZP3 binding assay, we compared ratios of the amount of ZP3 being eluted from the ZP1 beads against the starting amount of ZP3 for each ZP3 crude extract, using incompletely purified ZP1 and ZP3 samples. Therefore, we did not determine the precise amounts of ZP1 and ZP3 in the crude samples, while the differences among the starting amounts of ZP3 are normalized in the calculation of the ratios. To clarify that, the phrase “in the elution fractions to the crude ZP3-isoform fractions” was replaced with “in the elution fractions against the crude ZP3-isoform fractions” in lines 589 to 590 of both the Revised Manuscript with Track Changes and the Manuscript.

It is still unclear how ZP3 bind to ZP1 (i.e., structural details of ZP1–ZP3 interactions), and we cannot discuss how the contaminating ZP1 affect the ZP3 binding to the ZP1 beads.

-The binding assay is based on a quantification of bands obtained by immunoblot in order to identify the isoforms that best bind the ZP1 protein. As the fractions used contain several isoforms, do the authors know if all isoforms are equally recognised by the antibody? Also, the densitometry results sometimes seem to give inconsistent results. For example, in Figure 6C, band “b” in the crude extract appears visually much more intense than bands “a” and “c”, yet the densitometry values do not agree with this observation. In Figure 6G, band e in the crude extract appears much more intense (at least twice more intense) than bands “d” and “f”, but the densitometry values provided are close for all three bands. In the middle panel (elution), band “e” appears to be more intense than the band “f”, but here the densitometry results are the opposite.

**(Answer)**

All ZP3 isoforms must be equally recognized by the anti-ZP3 antibody being used in this study, because the antibody is a polyclonal one raised against the recombinant polypeptide of the ZP-C domain of chicken ZP3 that was produced using an *E. coli* system. Actually, the 2D-PAGE-separated ZP3 isoforms of F1 IPVLs from WL-G and commercial White-Leghorn hens were detected by CBB staining and immunoblotting using the anti-ZP3, respectively, without significant differences in signal intensity patterns (Figs 3A and 3B).

Densitometric values are calculated based on the sum of pixel intensities in the area of gel or blot images of protein bands or spots. Therefore, it sometimes happens that the results of densitometric analyses are inconsistent with the visual appearances of bands or spots, although the densitometric values are affected how to define the area of the signals. We performed the densitometric analyses against figures that the reviewer #2 pointed out, but there are not significant differences influencing the outcome between the re-analyzed results and the original ones used in the manuscript.

-There is no information on the number of repeats. Was this test repeated several times?

**(Answer)**

We did not repeat this test. However, we performed several incomplete versions of this test in addition to the more preliminary experiments based on far-western analyses against 2D-PAGE-separated ZP3 isoforms, and the results of them were fit well to the result of this test in summary.

-The authors conclude “these results showed that the ZP3 isoform 7 exhibited much higher binding affinity among ZP3 isoforms 2-8, although there might be possible synergistic effects of these ZP3 isoforms” (line 597-598). The data do not allow to be so affirmative about isoform 7 because fractions “c” and “f”, which according to the authors have the best binding capacity towards ZP1, also contain isoform 6. Why would isoform 7 bind ZP1 better than isoform 6? Furthermore, isoform 7 also appears to be a major component of fraction “e”. It is therefore difficult to conclude with certainty about the isoform that binds ZP1 best.

**(Answer)**

The phrase “(e.g., the ZP1 binding activity of the isoform 7 might be inhibited by the isoform 6 and be enhanced by the isoform 5)” was added after the sentence “These results showed that the ZP3 isoform 7 exhibited much higher ZP1 binding affinity among ZP3 isoforms 2–8, although there might be possible synergistic effects of these ZP3 isoforms.” in lines 599 to 601 of both the Revised Manuscript with Track Changes and the Manuscript.

For all these reasons, the results and conclusions of the ZP1-ZP3 binding test appear preliminary and need to be confirmed, either by using (more) pure ZP3 fractions or by using other methodological approaches.

**(Answer)**

The phrase “, and we need to confirm the results by using pure ZP3 isoform and ZP1 preparations” was added after the sentence “These results showed that the ZP3 isoform 7 exhibited much higher ZP1 binding affinity among ZP3 isoforms 2–8, although there might be possible synergistic effects of these ZP3 isoforms.” in lines 736 to 739 of both the Revised Manuscript with Track Changes and the Manuscript.

**Additional comments from the reviewer #2**:

1. The study was carried out on White Leghorn hens from two different origins, namely “commercial White Leghorn” and “long-term closed colony White Leghorn”. Why were two types of animals used in this study? In figure 3, the immunoblots from the “commercial White Leghorn” samples are compared with a coomassie gel from “long-term closed colony White Leghorn” animals. But a silver nitrate gel on the “commercial White Leghorn” is also shown in this figure. Why did the authors choose to compare densitometry results from samples of different origins? In panel B, it would be more rigorous to compare spots from samples from the same animals (e.g., the spots from immunoblots with the spots from the silver stained gel). Furthermore, the statistical analysis in Figure 3B does not mention the number of samples or animals used, nor the number of technical replicates.

**(Answer)**

We used the WL-G (the long-term closed colony of White-Leghorn breed) hens in addition to the commercial White-Leghorn hens to demonstrate that the isoelectric-point microheterogeneity of chicken ZP3 is not due to the potential genetic heterogeneities in commercial hens. In general, chicken especially the commercial hens possess genetic heterogeneities, although we can prepare abundant IPVL from their large ovarian follicles. That is mentioned in lines 676 to 678 of both the Revised Manuscript with Track Changes and the Manuscript, although not clearly described.

To make the reason using the WL-G line and the route of euthanasia for the WL-G lines, we corrected the sentence “Fresh ovary and genomic DNA of the long-term closed colony of White-Leghorn breed (WL-G) hens were provided” was corrected into “To analyze the genetically homogeneous line of hen, fresh ovary and genomic DNA of the long-term closed colony of White-Leghorn breed (WL-G) hens that were killed similarly by hands were provided” in lines 131 to 133 of the Revised Manuscript with Track Changes and lines 131 to 132 of the Manuscript.

2. The IPVL shown in Figures 1B, 1C, S1B, S1C, S1E, S1G (panels 2, 3, 6, 7) appear to have two sublayers. This two-layered structure is quite troubling. Is it an artefact or something real? This is not discussed in the results section.

**(Answer)**

The two-layered staining of the IPVL must be an artefact because of the low permeability of the IPVL against macromolecules such as antibodies. We therefore stained both surfaces of IPVL in the immunofluorescent staining (see Materials and Methods). Accordingly, we inserted the phrase “showing much lower permeability against macromolecules including antibodies than F2 IPVLs” in line 651 of both the Revised Manuscript with Track Changes and the Manuscript.

3. Lines 639-648: This part of discussion could be discussed with regards to the ultrastructural observations of the chicken zona radiata made by Wyburn in 1965: Wyburn GM, Aitken RN, Johnston HS. The ultrastructure of the zona radiata of the ovarian follicle of the domestic fowl. J Anat. 1965 Jul;99(Pt 3):469-84.

**(Answer)**

The phrase “, as partly mentioned in [14]” was added after the sentence “These results together with the observation that the F1 IPVLs are harder and easier to be detached from the ovarian follicles than the F2 IPVLs, suggest that the loose F2 IPVLs mature quickly into the F1 IPVLs in which the fibrous structures form tight associations with each other showing low permeability against macromolecules including antibodies, and the avian equivalent of mammalian TZPs that may traverse the F2 IPVL are closed before the ovulation in the F1 IPVLs.” in lines 648 to 653 of both the Revised Manuscript with Track Changes and the Manuscript.

4. The text needs to be proofread by a native English reader.

**(Answer)**

The reviewer #1 have read our last revised manuscript and answered “Yes” for the question “5. Is the manuscript presented in an intelligible fashion and written in standard English?” from the editor. Therefore, we think our manuscript has become readable enough for any English readers around the world through the last 3-times revisions.

**Minor remarks from the reviewer #2**:

-Figure 1: Scale values in µm are shown in the lower panels, but not in the upper panels. Please homogenise.

**(Answer)**

The scale values in µm are added in the upper panels in Figure 1 and Supplement figure 1.

-Figure 4: the asterisk is not described in the figure legend.

**(Answer)**

The asterisk in Figure 4 has already be shown in the figure legend in line 477 of both the Revised Manuscript with Track Changes and the Manuscript as “(*: P<0.01)”.

-Line 133: the abbreviation MEXT needs to be defined

**(Answer)**

The abbreviation MEXT was defined as “the Ministry of Education, Culture, Sports, Science and Technology (MEXT)” in line 134 of both the Revised Manuscript with Track Changes and the Manuscript.

-Line 211: "1% acrylamide" is probably not the right reagent for alkylation.

**(Answer)**

1% acrylamide are one of the reagents to be used for the cysteine alkylation. See the link below

<https://pubmed.ncbi.nlm.nih.gov/17484107/>

-Line 284: the use of "wt%" is not clear

**(Answer)**

The phrase “30 µl of the 75–80 wt% suspension” was replaced with “30 µl of suspension containing 75–80 wt% beads” in lines 285 to 286 of the Revised Manuscript with Track Changes and line 285 of the Manuscript.

**Corrections in the Reference list:**

Reference 15 was corrected with “Okumura H. Avian Egg and Egg Coat. In: Sasanami T, editor. Avian Reproduction: From Behavior to Molecules. Advances in Experimental Medicine and Biology. 1001. Singapore: Springer Singapore; 2017. p. 75-90.” in lines 838 to 840 of both the Manuscript with Track Changes and the Manuscript.

Reference 16 was corrected with “Nishio S, Okumura H, Matsuda T. Egg-Coat and Zona Pellucida Proteins of Chicken as a Typical Species of Aves. In: Litscher ES and Wassarman PM, editor. Extracellular Matrix and Egg Coats. Current Topics in Developmental Biology. 130. 20180326 ed: Elsevier; 2018. p. 307-29.” in lines 841 to 843 of both the Manuscript with Track Changes and the Manuscript.

Reference 26 was corrected with “D'Souza CA, Moscarello MA. Differences in susceptibility of MBP charge isomers to digestion by stromelysin-1 (MMP-3) and release of an immunodominant epitope. Neurochemical Research. 2006;31:1045–54.” in lines 876 to 878 of both the Revised Manuscript with Track Changes and the Manuscript.

Reference 35 was corrected with “Familiari G, Nottola SA, Macchiarelli G, Micara G, Aragona C, Motta PM. Human zona pellucida during in vitro fertilization: An ultrastructural study using saponin, ruthenium red, and osmium-thiocarbohydrazide. Molecular reproduction and development. 1992;32(1):51-61. doi: 10.1002/mrd.1080320109.” in lines 899 to 902 of both the Revised Manuscript with Track Changes and the Manuscript.
